# Effects of Creep on Shield Tunnelling Through Squeezing Ground

**DOI:** 10.1007/s00603-023-03505-x

**Published:** 2023-11-24

**Authors:** Thomas Leone, Alexandros N. Nordas, Georgios Anagnostou

**Affiliations:** ETH Zurich, Zurich, Switzerland

**Keywords:** Tunnel boring machine, Creep, Perzyna, Shield jamming, Lining overstressing, Standstill

## Abstract

The present work aims to improve the reliability of shield jamming and lining damage risk assessment in squeezing ground by analysing the effects of creep on the evolution of rock pressure over time. The study is based on numerical simulations of typical mechanised tunnelling processes, generally consisting of shield advance phases alternating with shorter or longer standstills for lining installation, maintenance, etc*.* A linear elastic—viscous plastic constitutive model based upon Perzyna’s overstress theory is employed, which considers the time-dependency of plastic deformations via a single viscosity parameter. The investigations demonstrate the following: (i) shield loading during advance increases with increasing viscosity under certain conditions, which contradicts the common perception in many existing works that creep is thoroughly favourable for shield jamming; (ii) creep is thoroughly unfavourable for shield loading during long standstills and long-term lining loading, due to the additional viscoplastic ground deformations manifested over time; (iii) the commonly adopted simplifying assumption of continuous excavation with the gross advance rate is adequate only where standstills are very short (e.g., for lining erection during the stop-and-go shield tunnelling process), but otherwise underestimates the shield loading, even in cases of regular inspection and maintenance standstills lasting only a few hours. Two application examples, the Fréjus safety gallery and the Gotthard Base tunnel, demonstrate the need to consider creep and the accuracy of modelling tunnel construction by a semi-discrete approach, where only the very short standstills for lining erection are considered via an average advance rate, but longer standstills are explicitly simulated.

## Highlights

- Assessment of the effect of creep on TBM shield loading.
- Assessment of the effect of creep on long-term lining loading.
- Analysis of a counter-intuitive, adverse effect of advance rate on shield loading.
- Discussion of alternative methods of considering excavation standstills.
- Development of a semi-discrete model for estimating shield loading during standstills.

## Introduction

In mechanised tunnelling through squeezing ground, jamming of the shield or damage to the lining may occur due to the development of substantial rock pressures on them (Ramoni and Anagnostou 2010a). Squeezing is often time-dependent, due to consolidation or creep (Anagnostou 2007). Time-dependency due to consolidation is relevant in medium- to low-permeability water-bearing ground, and is associated with the progressive dissipation of the excavation-induced excess pore pressures; its effects have been investigated *inter alia* by Graziani and Ribacchi (2001) and Ramoni and Anagnostou (2011b). The present paper focuses on the creep-induced time-dependency of squeezing.

General aspects related to the modelling of creep in tunnelling have been studied in numerous works, *e.g.* Corbetta (1990), Fritz (1981), Zienkiewicz et al. (1975) and Debernardi (2008). With a specific focus on shield tunnelling, several authors have investigated numerically the effect of creep on shield jamming during excavation (Swannell et al. 2016; Barla 2018; Barla et al. 2014; Hasanpour et al. 2015) and during tunnel boring machine (TBM) standstills (Mohammadzamani et al. 2019; Zhang and Zhou 2017). Other approaches are based on physical modelling (Arora et al. 2021a, 2021b, 2022) or artificial intelligence (Hou et al. 2022). Lining overstressing due to creep during advance (Hasanpour et al. 2015) and at steady state (De la Fuente et al. 2020) has also been examined, considering additionally the effect of backfilling (Liu et al. 2019), but it has attracted limited attention overall. In the above investigations there are two main limitations, as discussed hereafter.

First, most existing investigations are limited to specific tunnelling projects, and hence the applicability of their conclusions is limited. A common conclusion is that creep is always favourable for the shield and lining loading during advance in comparison with time-independent models, and even more so for higher advance rates (Swannell et al. 2016; Barla 2018; Hasanpour et al. 2015; Mohammadzamani et al. 2019; Zhang and Zhou 2017). This holds in most cases and is intuitively perceived as correct, since creep limits the extent of the squeezing deformations and pressures that develop during the short duration of excavation. Nonetheless, Ramoni and Anagnostou’s (2011b) investigations into the consolidation effects in a similar context indicated that the thrust force may paradoxically increase with decreasing permeability (an equivalent effect to that of increasing viscosity) under certain conditions, which raises the question of whether the same counter-intuitive behaviour may also occur in the case of creep. Considering the above, and the limited attention given to the aspect of long-term lining loading, there is scope for a more systematic investigation on the effects of creep which considers a wide range of practically relevant parameters.

Secondly, all existing works on tunnelling in ground exhibiting a time-dependent behaviour simulate the TBM operation as a continuous process, without explicitly considering the regular standstills that always occur during a TBM drive (very short standstills for lining erection in between successive TBM strokes and shift- or day-long ordinary standstills for cutterhead inspections, disk replacements, TBM maintenance, face mappings, etc.), or the very long extraordinary standstills (due to construction site holiday shutdowns or accidental incidents, such as cave-ins). Regular standstills are incorporated into continuous simulations by considering a gross advance rate that is considerably lower than the actual net advance rate during TBM strokes (e.g., Barla 2018, Barla et al. 2014, Hasanpour et al. 2015, Swannell et al. 2016); in some cases, this can be as low as 1–10 m/d (e.g., Ramoni and Anagnostou 2011b; Barla 2018; Hasanpour et al. 2015; Swannell et al. 2016), which one can only assume is intended to cover longer extraordinary standstills. The effect of standstills has been examined in only a few works and this in a simplified manner, basically considering a gross advance rate (Mohammadzamani et al. 2019; Zhang and Zhou 2017). Only Ramoni and Anagnostou (2011b) explicitly simulated a singular longer standstill upon completion of the excavation, but otherwise still incorporated the regular standstills during excavation via an average advance rate. As we will see later in this paper, the continuous simulation of the TBM advance may lead to a severe underestimation of the required thrust force, thus misleading the feasibility assessment of a TBM drive and the decision-making during design.

This paper aims to bridge the aforementioned knowledge gaps via a comprehensive and systematic numerical investigation into the following aspects: the effect of creep on the thrust force during advance or standstills and on the lining loads far behind the face under steady state conditions (i.e. after practically all time-dependent deformations have been completed), considering the range of viscosities relevant in tunnel engineering practice; the conditions under which the counter-intuitive behaviour (paradox) of higher thrust force during advance compared to that of time-independent problems is manifested (analogously to Ramoni and Anagnostou 2011b for consolidation); and the adequacy of considering the standstills by means of an equivalent average advance rate (hereafter referred to as “smearing” of standstills).

The paper proceeds in Sect. 2 with basic considerations on the constitutive modelling of creep in rock and the selection of an adequate model for the present investigations. Additionally, it explains the model behaviour and proposes a simple way of estimating viscosity in tunnelling boundary value problems. Subsequently, Sect. 3 introduces the computational model adopted in the numerical simulations of the construction process.

Section 4 presents a systematic investigation into the effect of creep on shield and lining loading for a wide range of parameters relevant in tunnelling. Additionally, it demonstrates that disregarding creep, and thus assuming for simplicity that squeezing occurs instantaneously upon excavation, is not necessarily a conservative simplification, but may instead result in an underestimation of the pressure developing upon the shield, and thus of the necessary thrust force during TBM advance. This apparent paradox is explained as the result of two competing effects of the slower deformation development: the ground on the one hand establishes contact with the shield later, which is favourable, but on the other hand experiences less stress relief ahead of the face, which is unfavourable. The latter is also the reason that the long-term lining loading is always higher in the presence of creep.

Section 5 addresses the question of excavation standstill modelling, by comparing the adequacy of various approaches. It shows that considering an average advance rate is adequate only for the stop-and-go process of shield tunnelling, where the very short standstills required for lining erection frequently and regularly alternate with the short net advance phases during TBM strokes; conversely, shift- or day-long standstills, such as those regularly required for inspections and maintenance works, and longer extraordinary standstills must be considered explicitly. It also introduces a “semi-discrete” approach, where only the standstills during the stop-and-go process are smeared, but the longer standstills are simulated explicitly.

Finally, Sect. 6 demonstrates the effects of creep, as well as the importance of an adequate modelling of standstills and the accuracy of the proposed semi-discrete simulation method, via two application examples concerning the Fréjus safety gallery and the Gotthard base tunnel.

## Constitutive Modelling

A broad range of constitutive models has been adopted in existing works, encompassing different combinations of mechanical (time-independent) and rheological (time-dependent) counterparts. Table 1 provides an overview of some of the most widely employed models in the literature.

**Table 1:** Overview of constitutive models with rheological behaviour

*Constitutive model*	SHELVIP^a^	3SC^b^	CVISC^c^	Lemaitre^d^	MC-Perzyna^e^	Ghaboussi model^f^
*Mechanical analogy*						
*Nr. of parameters*	11	9	9	8	6	5
*Yield condition*	Drucker Prager	Mohr Coulomb	Mohr Coulomb	Von Mises, Drucker Prager^g^	Mohr Coulomb	–
*Elastic time dependency*	No	Yes	Yes	No	No	Yes
*Plastic time dependency*	Yes	Yes	No	Yes	Yes	No
*Time-dependency theory*	Overstress theory^h^	Newton’s viscosity law and Overstress theory^h^	Newton’s viscosity law	Overstress theory^h^	Overstress theory^h^	Newton’s viscosity law
*Deviatoric/volumetric time-dependency*	Deviatoric	Deviatoric	Deviatoric	Deviatoric	Deviatoric	Deviatoric
*Tunnel projects*	Lyon Turin^i^ Kishanganga^j^	Lyon Turin^k^	Xiangjiaba^l^, Lyon Turin^m^ Raticosa^n^, Fréjus ^o^	Raticosa^n^	–	–
*Commercial FE software*	–	–	FLAC 2D|3D^p^	–	–	–
*Predicted creep test stages*	Primary and secondary	Primary and secondary	Primary and secondary	Primary and secondary	Secondary	Primary

^a^Debernardi (2008)

^b^Sterpi and Gioda (2009)

^c^Burgers (1935), Itasca (2019)

^d^Boidy et al. (2002)

^e^Zienkiewicz and Cormeau (1974); Corbetta (1990);Bernaud (1991)

^f^Ghaboussi and Gioda (1977)

^g^Pellet (2004)

^h^Perzyna (1966)

^i^Debernardi (2008);Barla et al. (2012)

^j^Barla et al. (2014)

^k^Barla et al. (2012)

^l^Y.Zhang et al. (2015)

^m^Barla et al. (2008);Pellet (2009)

^n^Bonini et al. (2007)

^o^De la Fuente et al. (2020)

^p^Itasca (2019)

In the present work, which analyses creep effects based on numerical parametric investigations, the main criteria for model selection are its formulation simplicity, since models with fewer parameters enable a better qualitative interpretation of the results, and the consideration of time-dependency in the plastic regime, where squeezing deformations mainly occur. The only model readily implemented in a commercial FE code is CVISC (Burgers 1935; Itasca 2019); however, it fulfils neither of the above criteria and has also been shown to predict a lining pressure equal to the in-situ stress at steady state conditions (De la Fuente et al. 2020), which contradicts field experience. Although this is not important for modelling the processes in the vicinity of the advancing tunnel face, it is important for the investigation of lining overstressing at steady state conditions considered in the present work. From the remaining models, SHELVIP, 3SC and Lemaitre have more complex multiparametric formulations, while the Ghaboussi model, although simpler, only considers viscoelasticity (*cf.* Table 1).

Considering the above and the objective of the paper, which is to improve the fundamental understanding of time-dependent effects on shield jamming and lining overstressing, it is sufficient to consider the simplest possible constitutive model, so as to enable an easier qualitative interpretation of the computational results. An isotropic, linear elastic and viscous perfectly plastic model is thus adopted, based on Hooke’s law and Bingham’s rheological model (Table 1), with a Mohr–Coulomb (MC) yield condition and a non-associated viscoplastic flow rule after Perzyna’s (1966) overstress theory. The model, hereafter referred to as “MC-Perzyna”, considers solely plastic time-dependency by resolving the strain rate into an elastic part and an inelastic part that incorporates the combined viscous and plastic effects:

1 $${\dot{\mathbf{\varepsilon }}} = {\dot{\mathbf{\varepsilon }}}^{el} + {\dot{\mathbf{\varepsilon }}}^{vp} .$$

The elastic part ${\dot{\mathbf{\varepsilon }}}^{el}$ depends linearly on the stress rate, according to Hooke’s law, while the inelastic part is given by the following expression:

2 $${\dot{\mathbf{\varepsilon }}}^{vp} = \frac{{f_{i} }}{\eta }\frac{\partial g}{{\partial {{\varvec{\upsigma}}}}},$$

where *f*_*i*_ is the MC yield function, g the plastic potential function and *η* the viscosity, Eq. 2 involves the assumption that the inelastic strain rate depends on the excess stresses lying above the yield surface (Perzyna 1966).

The model formulation encompasses in total only 6 parameters: 2 elasticity constants (Young’s modulus *E*, Poisson’s ratio *ν*), 3 plasticity constants (angle of internal friction *ϕ*, cohesion *c*, angle of dilation *ψ*), and the viscosity *η* that governs the viscoplastic behaviour. It is noted that, although an isotropic model cannot capture the non-uniformity of rock deformations and pressure over the tunnel circumference, it can still be used to obtain reasonable estimates for anisotropic rocks if necessary, by considering an appropriate set of equivalent parameters (Mezger 2019). Systematically incorporating anisotropic effects in the context of the parametric studies conducted in the present work would anyway increase their already substantial size and the complexity of the computational model, as it would require 3D computations that consider different combinations of anisotropy plane orientations, as well as of stiffness, strength and viscosity anisotropies.

The MC-Perzyna model has been implemented in Abaqus^®^ (Dassault Systèmes 2018) by the first author as a user-defined material (UMAT) subroutine. The key aspects of its formulation and numerical implementation are outlined in the Appendix.

While engineers have some experience in estimating common material parameters, such as the modulus of elasticity, compressive strength, or friction angle, this is less true for the parameters that determine the rate of creep, *i.e*. the viscosity *η* for the constitutive model adopted here. If experiences of the temporal development of squeezing or results of field measurements from adjacent underground openings are available, these could be used to back-calculate the viscosity. As an aid for a simplified back-analysis, consideration is given in the sequel to the plane-strain, rotationally symmetric problem of a deep, cylindrical, and uniformly supported tunnel of radius *R*, crossing homogeneous rock subjected to a uniform and hydrostatic in-situ stress field. The tunnel boundary is instantaneously unloaded from the in-situ stress (σ_0_) to zero support pressure (*σ*_*R*_ = 0) and the radial displacement (*u*) development over time is monitored.

The MC-Perzyna model predicts a purely elastic instantaneous displacement that agrees with the well-known analytical solution of Kirsch (1898). If yielding occurs in the ground around the tunnel, viscoplastic displacements start taking place at a rate governed by the viscosity *η*. For the borderline case* η* = 0 (time-independent model) all plastic displacements occur instantaneously, and the response is described by the well-known elastoplastic solution (see, *e.g*., Anagnostou and Kovári 1993). For the other borderline case *η* → ∞ (infinitely viscous model) no viscoplastic displacements occur within all practically relevant time periods. In intermediate cases 0 < *η* < ∞ the displacement increases at a decreasing rate, and at steady state (*i.e.,* after the viscoplastic deformations have been practically completed) tends asymptotically to the elastoplastic solution.

The displacement of the considered unsupported tunnel depends in general on all independent problem parameters, specifically the initial stress σ_0_, the tunnel radius *R*, the time *t*, the viscosity *η* and the other five material constants:

3 $$u = f\left( {\sigma_{0} ,R,t,\eta ,E,v,f_{c} ,\phi ,\psi } \right).$$

Based upon dimensional analysis, and considering that the elastoplastic deformations are inversely proportional to the Young’s modulus (Anagnostou and Kovári 1993), Eq. 3 can be written in the following nondimensional form (see, e.g., Ramoni and Anagnostou 2010b), which expresses the normalised displacement (left hand side term) as a function of the normalised time (first right hand side term):

4 $$\frac{u\;E}{{R\;\sigma_{0} }} = f\left( {\frac{tE}{\eta },\;\frac{{f_{c} }}{{\sigma_{0} }},\phi ,\;\psi ,\;\nu } \right).$$

One can readily verify that the time required to achieve a given percentage, *e.g.,* 95%, of the time-dependent displacement increment is solely a function of the material constants:

5 $$\frac{{t_{95} E}}{\eta } = f\left( {\frac{{f_{c} }}{{\sigma_{0} }},\phi ,\;\psi ,\;\nu } \right).$$

**Fig. 1:**
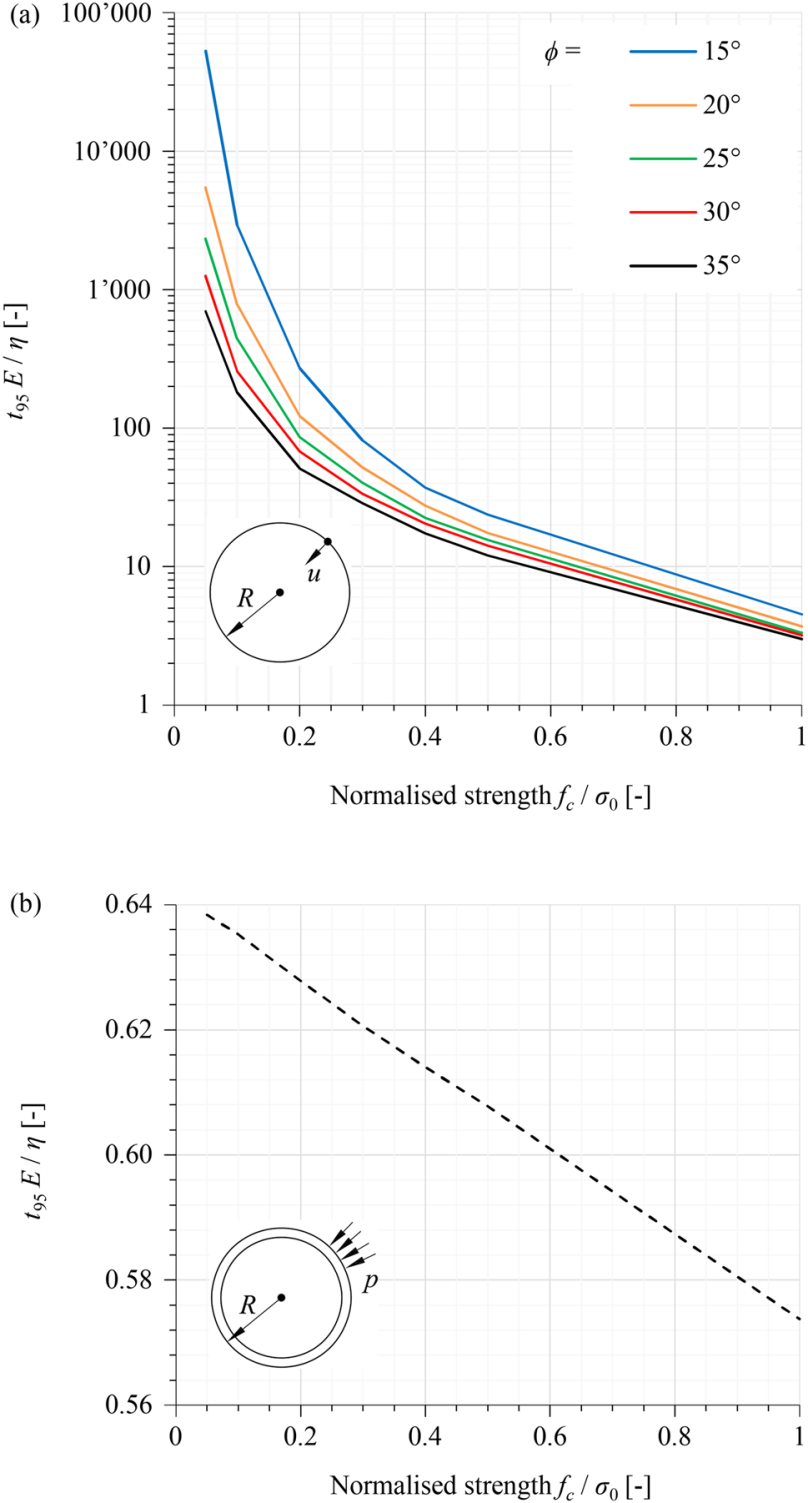
Normalised time *t*_95_ as function of the strength parameters: **a** unsupported tunnel; **b** tunnel with rigid support (*ν* = 0.25, *ψ* = *ϕ*–20°)

This relationship was quantified numerically by means of rotationally symmetric plane strain computations and is presented graphically in Fig. 1a. A similar relationship is obtained in the case where a rigid support is instantaneously applied over the tunnel boundary upon unloading, as shown in Fig. 1b, where *t*_95_ denotes the time required for 95% of the final pressure to develop on the lining.

For the case of an unsupported tunnel, *t*_95_ decreases with increasing *ϕ* and *f*_*c*_/σ_0_ (Fig. 1a), since higher strength parameters result in smaller total viscoplastic deformations, and thus a shorter time for attaining 95% of their final value. A similar trend is observed in the case of a rigid support, but *t*_95_ is practically constant. Interestingly, *t*_95_ is smaller by at least one order of magnitude than the case of an unsupported tunnel, which indicates that providing support to the ground, and thus fully constraining the viscoplastic deformations, accelerates the stress relief that would otherwise occur through said deformations. Practically relevant cases, i.e., a lining of finite stiffness providing a finite support pressure after a certain ground pre-deformation, may lie between the curves of the two cases in Fig. 1.

The diagrams in Fig. 1 allow the viscosity *η* to be determined in a simple manner, based upon the observed time-development of deformations (which provides an indication as to *t*_95_) and the estimated values of the strength parameters (*f*_*c*_, *ϕ*), Young's modulus *E*, and initial stress σ_0_.

## Computational Model of TBM Advance

**Fig. 2:**
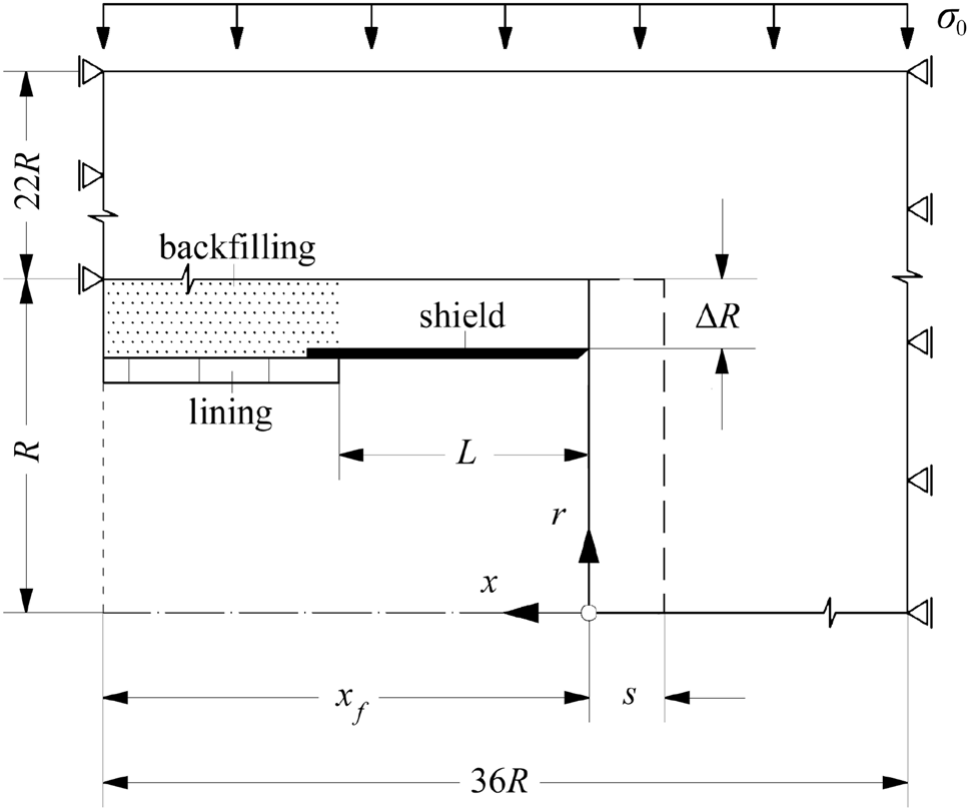
Computational domain (not to scale)

For the numerical investigations presented in the next sections, an axisymmetric Finite Element (FE) model has been developed in Abaqus^®^ (Dassault Systèmes 2018) that simulates the mechanised excavation and lining installation sequence step-by-step (see, e.g., Franzius and Potts 2005), as well as the subsequent transient processes during a standstill of arbitrary duration (Fig. 2). In addition to the trivial assumptions underlying rotationally symmetric tunnel analyses (e.g., a uniform and hydrostatic in-situ stress field, homogeneous and isotropic rock, etc.; *cf.* Sect. 2), the model assumes negligible TBM weight, and thus uniform tunnel support and overcut around the shield, as well as uniform backfilling around the segmental lining.

At each numerical excavation step, part of the ground is removed ahead of the advancing tunnel face (round length *s*) and an equal part of lining is installed immediately behind the shield tail. The model considers the actual times required for excavating the ground with the TBM cutterhead and for installing one ring of the segmental lining and allows each excavation step to be simulated either discretely (short phases of continuous advance alternating with short standstills; the so-called “stop-and-go”) or continuously (continuous advance at a smeared rate and no standstill).

The tunnel face is considered unsupported. The shield of length *L* is modelled with non-linear radial springs, which consider no loading (zero stiffness) for rock convergences smaller than the overcut Δ*R*, and a linear elastic stiffness *K*_*s*_ for the portion of convergences that exceeds Δ*R*. The lining is modelled with elastic radial springs of stiffness *K*_*l*_, assuming that it is in direct contact with the ground immediately upon installation due to backfilling. The consideration of distinct shield and lining installation points enables capturing the ground unloading immediately behind the shield tail and its reloading over the lining (Fig. 2). For more details the reader is referred to Ramoni and Anagnostou (2010b). The MC-Perzyna model (Sect. 2) is adopted for the ground.

**Fig. 3:**
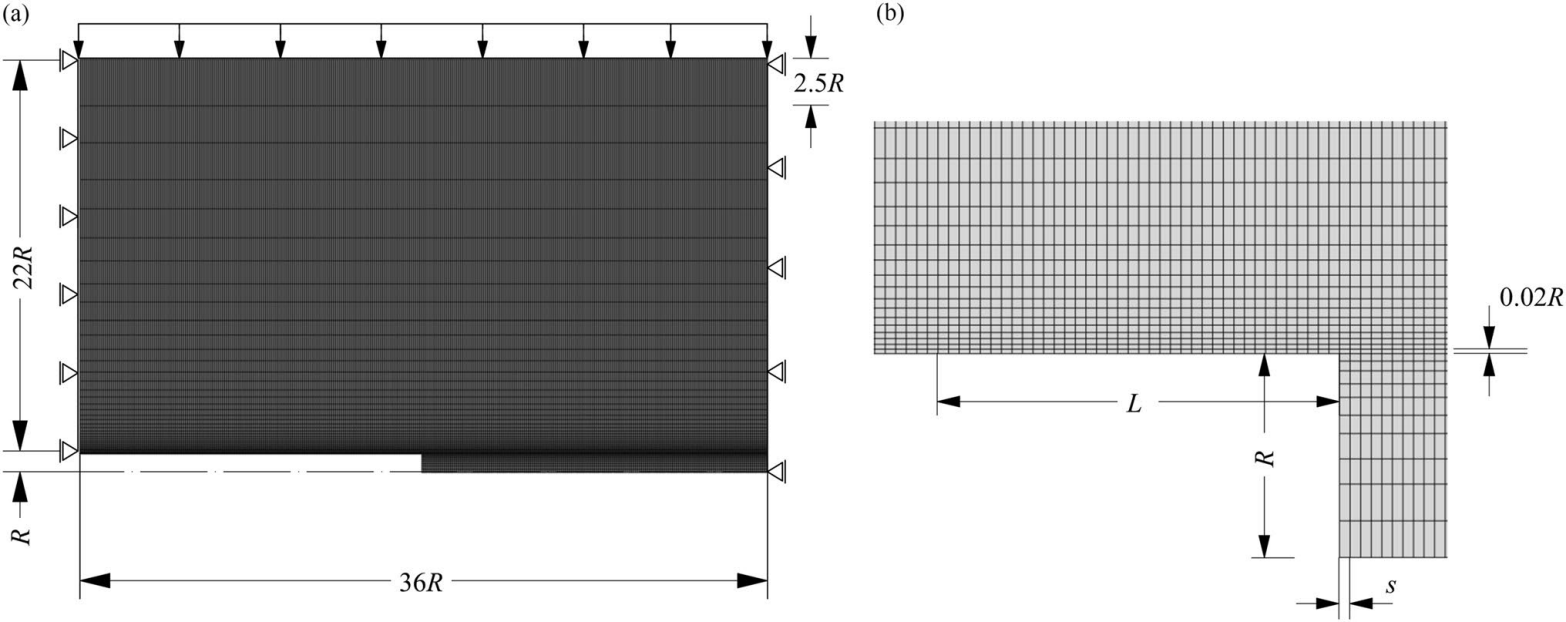
Finite element mesh: **a** global configuration; **b** detail in the vicinity of the tunnel face

For the spatial discretisation of the computational domain, a structured mesh encompassing 36822 4-noded, linear, quadrilateral, axisymmetric finite elements (FEs) have been employed, as shown in Fig. 3. In the radial direction (*r*), the element size varies with an exponential bias between 0.02*R* at the tunnel boundary and 2.5*R* at the upper far field boundary. Along the longitudinal *x*-axis of the tunnel, the element size is constant and equal to the round length *s* (Figs. 2, 3). Introducing a single FE within each round length enables eliminating the saw-shaped fluctuations in the distribution of the radial rock pressure that are typically observed in step-by-step simulations (Cantieni and Anagnostou 2009). The round length *s* is taken as 0.25 m, except for cases where a specific length of the segmental lining rings is considered; in such cases, *s* is taken equal to the ring length. The various round lengths considered in different simulations are always sufficiently small to ensure enhanced prediction accuracy (Franzius and Potts 2005).

The developed computational model provides *inter alia* the longitudinal profile of the ground pressure *σ*_*R*_ (*x*) acting upon the shield and the lining. The thrust force required for overcoming shield skin friction can then be evaluated from the following expression:

6 $$F_{r} = \mu \;2\pi R\;\int\limits_{0}^{L} {\sigma_{R} \left( x \right)dx = } \mu \;2\pi R\,L\,\overline{\sigma }_{R} ,$$

where *μ* is the shield skin friction coefficient, and $\overline{\sigma }_{R}$ the mean pressure over the shield. In each numerical simulation, the excavated tunnel stretch is considered sufficiently long for the thrust force to become constant and the standstill stage is initiated thereafter.

## Effects of Creep on Rock Pressure

The effects of creep on the shield and lining loading are numerically investigated in this section using the computational model presented in Sect. 3, considering a continuous excavation at an average advance rate of 20 m/d followed by a standstill and the parameters given in Table 2.

**Table 2:** Parameters considered in numerical computations of Sects. 3, 4, 5

Ground			
Young’s Modulus	*E*	[MPa]	1000
Poisson’s ratio	*ν*	[–]	0.25
Angle of internal friction	*ϕ*	[°]	25
Dilatancy angle	*ψ*	[°]	5
Uniaxial compressive strength	*f*_*c*_	[MPa]	1.25
Viscosity	*η*	[MPa^.^d]	variable
In-situ stress	σ_0_	[MPa]	25
TBM			
Boring radius	*R*	[m]	5
Shield length	*L*	[m]	10
Annular gap (overcut)	Δ*R*	[cm]	12.5
Shield radial stiffness	*K*_*s*_	[MPa/m]	2000^a^
Shield skin friction coefficient	*µ*	[–]	0.1, 0.15^b^
Boring force	*F*_*b*_	[MN]	17
Lining			
Radial stiffness	*K*_*l*_	[MPa/m]	100^a^
Computational model			
Round length	*s*	[m]	0.25
Model size in radial direction	*R*_*m*_	[m]	110
Model size in longitudinal direction	*L*_*m*_	[m]	180

^a^Conservative assumption after Ramoni and Anagnostou (2010b)

^b^Sliding and static friction coefficients with lubricated shield extrados after Ramoni and Anagnostou (2011a)

### Basic Behaviour

First, the basic behaviour of the system is examined, with the focus on the rock-shield-lining interaction. Three cases of viscosity are considered: a very low viscosity, characterising practically time-independent behaviour (*η* = 0.5 MPa^.^d, *t*_95_ = *ca.* 30 s–1 day in the boundary value problems of Fig. 1b and a, respectively); an intermediate viscosity, at which steady state is reached within hours or months (*η* = 200 MPa^.^d*, t*_95_ = *ca.* 3 h–14.5 months after Fig. 1b and a, respectively); and a high viscosity, which would result in time-dependent deformations for several months or years (*η* = 300,000 MPa^.^d, *t*_95_ = *ca.* 6.5 months–1830 years after Fig. 1b and a, respectively).

**Fig. 4:**
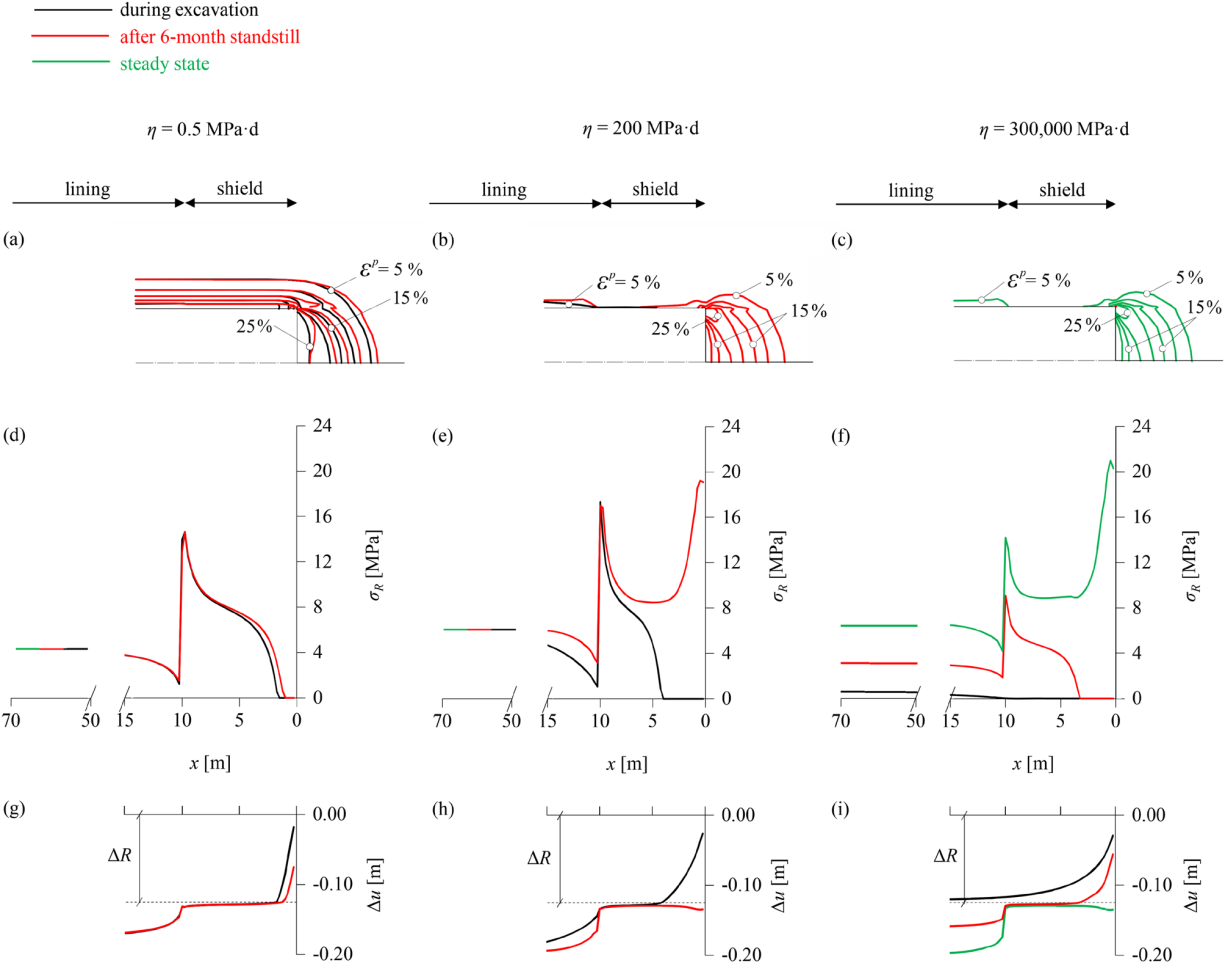
Contour-lines of plastic strain for *η* = 0.5 MPa^.^d (**a**), *η* = 200 MPa^.^d (**b**) and *η* = 300,000 MPa^.^d (**c**); longitudinal rock pressure distribution for *η* = 0.5 MPa^.^d (**d**), *η* = 200 MPa^.^d (**e**) and *η* = 300,000 MPa^.^d (**f**); and longitudinal convergence profile for *η* = 0.5 MPa^.^d (**g**), *η* = 200 MPa^.^d (**h**) and *η* = 300,000 MPa^.^d (**i**) during advance (black), after a 6-month standstill (red) and at steady state (green) (advance rate 20 m/d; other parameters: Table 2)

Figure 4a–c show the equivalent plastic strain *ε* ^*p*^ (magnitude of plastic strain vector) around the tunnel heading during advance (black lines), after a 6-month standstill (red lines), and at steady state (green lines) for the three viscosity cases. In the low-viscosity case (Fig. 4a) time-dependency is negligible; all plastic deformations occur practically simultaneously with excavation and remain practically constant over time (green and red lines coincide). Conversely, in the high-viscosity case (Fig. 4c), where time-dependency is very pronounced, plastic deformations in the immediate vicinity of the tunnel are negligible during excavation and within the 6 months of the standstill, and only occur later. In the intermediate case (Fig. 4b), plastic deformations occur partially during excavation and partially within the 6-month standstill, mainly in the ground ahead of the face, and remain constant thereafter (green and red lines coincide).

The occurrence of plastic deformations, or the lack thereof, is reflected in the longitudinal rock pressure and convergence profiles (Fig. 4d–f and g–i, respectively). In the low-viscosity case, the ground deformations during excavation, and thus the contact area with the shield (Fig. 4g) and the exerted pressure (Fig. 4d), are the greatest amongst the three cases, and increase only slightly during the standstill. Conversely, in the high-viscosity case there is no contact between ground and shield during excavation, as elastic convergences do not exceed the overcut Δ*R* (Fig. 4i), while during the standstill viscoplastic deformations develop extremely slowly and exert a small pressure on the shield (Fig. 4f). For the intermediate viscosity, contact between shield and ground already occurs during excavation (Fig. 4h) over a smaller area compared to the low-viscosity case; however, the prevention of additional viscoplastic deformations during the 6-month standstill by the static shield results in the most unfavourable shield loading among the three cases (Fig. 4e). The local pressure peak close to the unsupported face, which has also been observed in the investigations of Ramoni and Anagnostou (2011b) into the effects of consolidation on shield jamming, is associated with plastic yielding of the core ahead of the face, and has been shown to vanish in the presence of a sufficient face support.

These results clearly indicate that the interplay between standstill duration and viscosity is the most critical aspect. These effects are investigated in the sequel in more detail.

### Time-Development of Shield Loading During Standstills

**Fig. 5:**
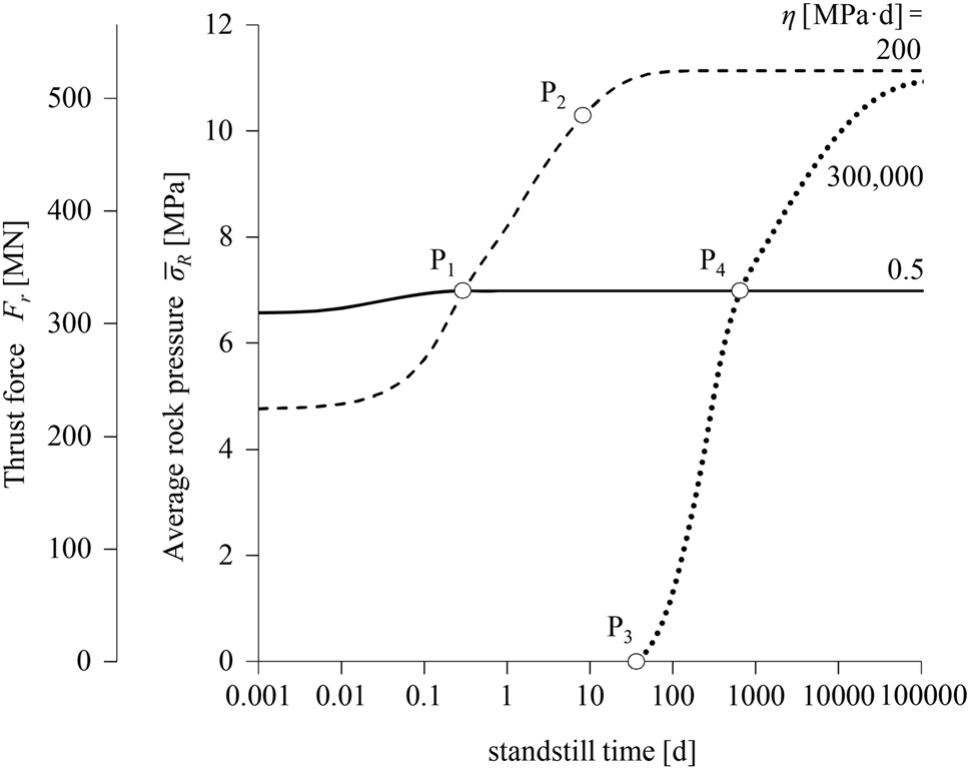
Increase in the thrust force and in the average rock pressure developing on the shield during a standstill (advance rate 20 m/d; other parameters: Table 2)

Figure 5 shows the increase in the average rock pressure developing upon the shield ( $\overline{\sigma }_{R}$) during the standstill and, on the second vertical axis, the corresponding thrust force required to overcome shield skin friction (*F*_*r*_; Eq. 6). For the low viscosity (solid line), the behaviour is almost time-independent and steady state is reached practically already during excavation; the thrust force increases only slightly and only for a few hours, remaining practically constant thereafter. This is also reflected in the rock pressures and convergences in Fig. 4d and g, where the red lines which hold after a 6-month standstill are very close to the black lines which hold for the conditions during excavation. For the high viscosity (dotted line), the annular gap remains open during excavation (see black line in Fig. 4i); the ground establishes contact with the shield during a standstill, after about 40 days (point P_3_), and subsequently exerts an increasing pressure which, after about 500 days, exceeds the pressure that would develop in the case of practically time-independent behaviour (point P_4_); however, this case is irrelevant from the practical engineering viewpoint since standstill durations are typically much shorter. Nevertheless, a similar behaviour can be observed for the moderate viscosity too (dashed line). In this case, the instantaneous thrust force is smaller than with the time-independent model (*η* = 0.5 MPa^.^d) but reaches the same value very rapidly during a standstill (within less than 1 day; point P_1_) and a value about 50% higher after almost 10 days (point P_2_). This case is therefore the most critical from a practical engineering viewpoint.

In conclusion, assuming that the plastic deformations develop instantaneously is in no way a conservative assumption: Models that disregard creep may overestimate the shield loading during excavation but considerably underestimate the rock pressure developing during (even short) standstills.

### Effect of Viscosity on Shield Loading

**Fig. 6:**
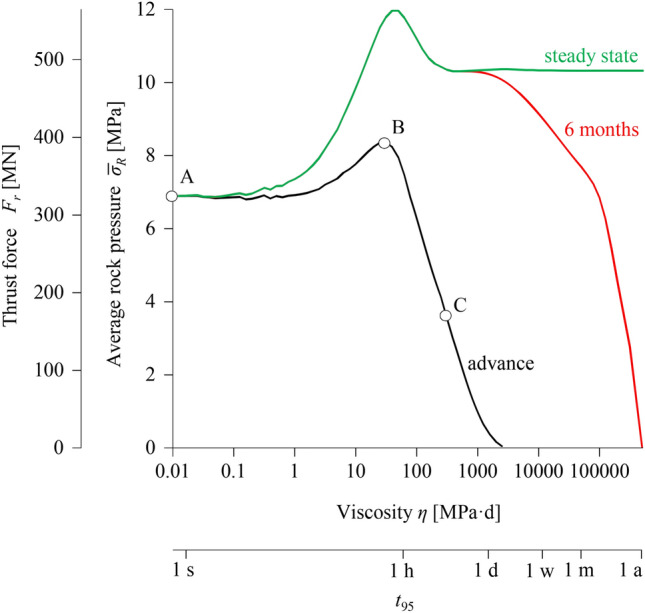
Thrust force and average rock pressure developing on the shield as a function of the viscosity *η* (advance rate 20 m/d; other parameters: Table 2)

Figure 6 shows the effect of viscosity on the average rock pressure developing upon the shield and on the thrust force during advance, after a 6-month standstill and at steady state. The second abscissa axis shows the time required to reach practically steady state conditions in the plane strain problem of a rigidly supported tunnel of Fig. 1b, thus providing a sense of the creep intensity for any given *η*-value. The rock pressure is identical in all three time-instances for very small viscosities, where the behaviour is practically time-independent. The curves separate thereafter, since additional viscoplastic deformations that occur over time increase the pressure on the shield; this effect is more pronounced in the case of intermediate and high viscosities, for which the conditions during advance are far away from the ones at steady state. With increasing viscosity, the rock pressure increases to a maximum value and thereafter decreases, becoming zero (no contact between ground and shield) for sufficiently large values, both during excavation and after a standstill. Conversely, the steady-state curve remains constant after a point, since contact will eventually occur after a sufficiently long time, even for extremely high viscosity values. The 6-month and the steady-state curves coincide for viscosities up to about 1000 MPa^.^d, as in this range steady state is reached in less than 6 months.

### Analysis of the Counter-Intuitive Behaviour

**Fig. 7:**
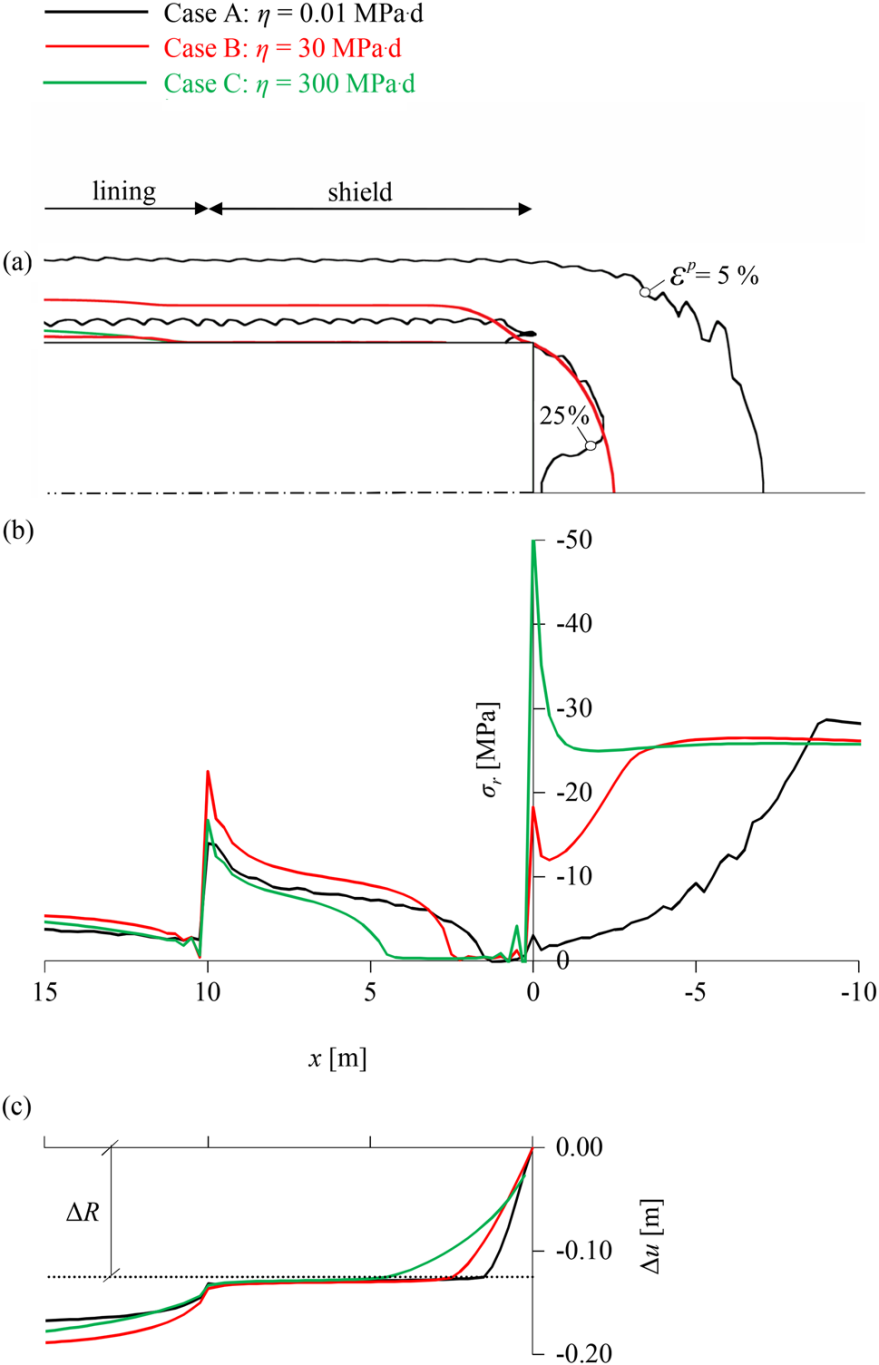
Contour-lines of plastic strain (**a**), longitudinal rock pressure and radial stress distribution (**b**), and longitudinal convergence profile (**c**) during advance for *η* = 0.01 MPa^.^d (black), *η* = 30 MPa^.^d (red) and *η* = 300 MPa.^.^d (green) (advance rate 20 m/d; other parameters: Table 2)

The peak in the relationship between rock pressure and viscosity is counter-intuitive at first glance. One would expect the pressure to monotonically decrease with increasing viscosity, due to delayed occurrence of viscoplastic deformations. This seemingly paradoxical behaviour is explained with reference to Fig. 7, which shows the equivalent plastic strains (Fig. 7a) and the longitudinal rock pressure and convergence profiles (Fig. 7b, c, respectively) during advance for cases A, B and C annotated on Fig. 6.

Figure 7 demonstrates two counteracting effects of increasing viscosity: (i), the annular gap closes later, and hence the contact area between ground and shield decreases (Fig. 7c), whereas, (ii), stress relief in the ground ahead of the face is less pronounced (Fig. 7b) due to the smaller plastic deformations (Fig. 7a), and hence the pressure transferred to the shield when excavating the ground is higher. In case A, where the behaviour is practically time-independent, contact occurs closer to the face than in cases B and C (Fig. 7c), but the relaxation of the ground ahead of the face is much greater, as the plastic zone is much more extended (Fig. 7a) and the radial stresses are much lower (Fig. 7b). In case B, the contact point moves further behind the tunnel face than in case A (Fig. 7c); however, the stresses exerted on the shield are higher because the pre-excavation stresses are higher (Fig. 7b), and the contact occurs close enough to the face for this influence to be relevant. In case C with the highest viscosity, the pre-excavation stresses ahead of the face are even higher than in case B (Fig. 7b) but contact is established too far behind the face (Fig. 7c) for the average shield loading to be influenced, and the latter is thus lower than in case B.

In conclusion, it can be said that for the range of lower viscosities (between points A and B), the effect of the pronounced stress relief (ii) dominates over that of the contact area (i), whereas the opposite holds in the range of higher viscosities (between points B and C). This interplay produces the counter-intuitive behaviour, which has not been reported in the literature thus far, but is unfavourable for shield jamming during advance and also later on (Fig. 6).

### Effect of Viscosity on Lining Loading

**Fig. 8:**
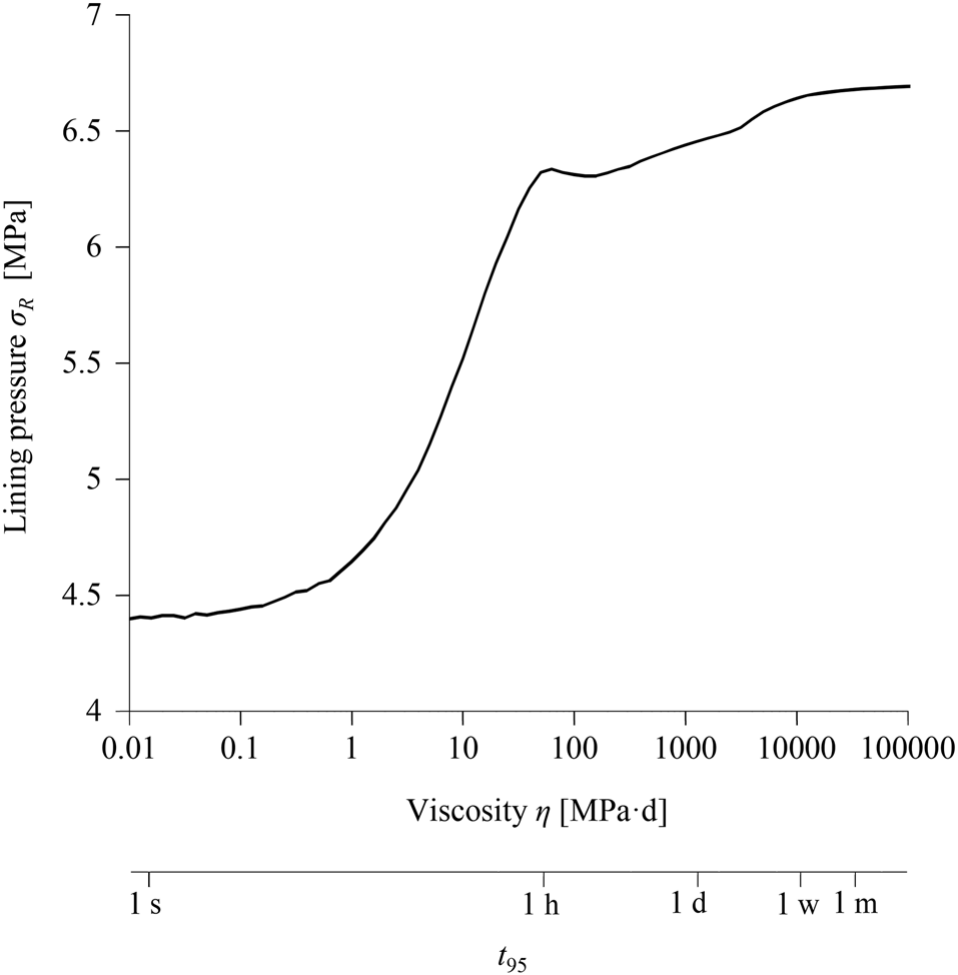
Steady-state lining pressure at a distance of 5 diameters behind the tunnel face as a function of the viscosity *η* (advance rate 20 m/d; other parameters: Table 2)

Figure 8 shows the effect of viscosity on the pressure that develops on the lining at a cross-section far behind the face (distance 10*R*) at steady-state conditions. With increasing viscosity, the lining pressure increases practically monotonically and reaches a maximum value at very high viscosities, for which the ground response to tunnelling is practically elastic in the vicinity of the advancing heading. The higher the viscosity, the lower will be the short-term lining load, the lesser will be the stress relief that the ground experiences before lining installation, and the greater will be the stress relief of the ground over time that will be accommodated by the lining (see green lines of steady-state lining pressure in Fig. 4d–f). This effect is qualitatively similar in the case of consolidation: a low permeability (equivalent to high viscosity) is more unfavourable for the final lining loading since the excess pore pressure dissipation and the resulting ground relaxation due to consolidation happen long after the excavation (Ramoni and Anagnostou 2011b).

## Modelling of Standstills

**Fig. 9:**
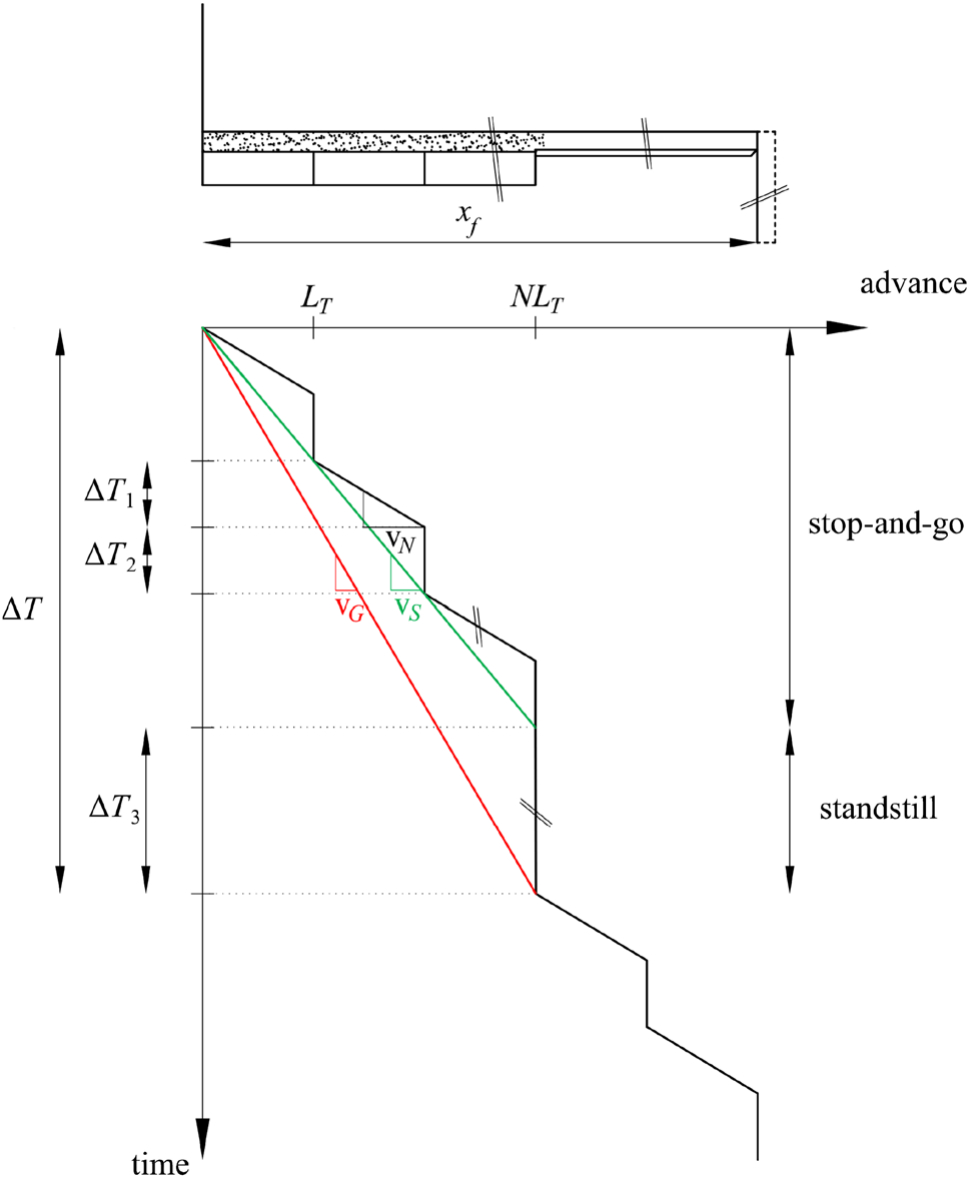
Parameters for discrete, semi-discrete and continuous simulations of a full TBM operation cycle consisting of several stop-and-go cycles followed by one standstill

Regular TBM operation can be idealised as a discrete process of periodically repeated excavation cycles of duration Δ*T*, as shown in Fig. 9. Each cycle starts with a stop-and-go phase, where continuous boring takes place over a period Δ*T*_1_ = *L*_*T*_ / v_*N*_, *L*_*T*_ being the length of one lining ring and v_*N*_ the net advance rate, followed by a short standstill for the erection of the lining ring over a period ΔT_2_. The installation of *N* lining rings is followed by an ordinary standstill for cutterhead inspections, disk replacements, maintenance, face mappings, etc. over a period Δ*T*_3_, which typically ranges between a few hours and 1–2 days. The total duration of one excavation cycle can thus be expressed as Δ*T* = *N* (Δ*T*_1_ + Δ*T*_2_) + Δ*T*_3_. Exceptional incidents, e.g., cave-ins, may also occur at any point during the advance (“extraordinary standstills”), forcing the TBM to remain at standstill for much longer periods.

As mentioned in the Introduction, all existing works on ground exhibiting time-dependent response to tunnelling simulate the TBM advance as continuous (e.g., Ramoni and Anagnostou 2011b; Barla et al. 2014; Barla 2018; Hasanpour et al. 2015; Mohammadzamani et al. 2019; Zhang and Zhou 2017, etc*.*), which is equivalent to a smearing of the full excavation cycle considering a gross advance rate v_*G*_ (red line in Fig. 9). The latter is considerably lower than the net advance rate v_*N*_, according to the following expression:

7 $$\text{v}_{G} = \text{v}_{N} \left( {1 + \frac{{\Delta T_{2} }}{{\Delta T_{1} }}} \right)^{ - 1} \left( {1 - \frac{{\Delta T_{3} }}{\Delta T}} \right)$$

An intermediate approach to considering the gross advance rate is to smear only the very short standstills for lining erection during the stop-and-go phase (Δ*T*_2_), considering an equivalent advance rate v_*S*_ (green line in Fig. 9), with

8 $$\text{v}_{S} = \text{v}_{N} \left( {1 + \frac{{\Delta T_{2} }}{{\Delta T_{1} }}} \right)^{ - 1} ,$$

but otherwise explicitly simulate the longer ordinary standstills (Δ*T*_3_). This so-called “semi-discrete” approach was also adopted in the computations in Sect. 4.

In the following, the accuracy of the simplified method that considers continuous excavation with the gross advance rate and of the proposed semi-discrete simulation will be assessed in relation to a formally correct discrete simulation, based on the predicted required thrust force. The total required thrust force during TBM advance consists in general of a boring force and a friction force (Eq. 6), which is evaluated considering the sliding shield skin friction coefficient. For the TBM restart after a standstill only the friction force needs to be considered, which, however, must be evaluated for the higher static shield skin friction coefficient. This distinction is only possible in discrete simulations; in semi-discrete and continuous simulations, the thrust force is assumed equal to the maximum between its values during advance and during TBM restart after a standstill. For the results discussed hereafter, a typical value of 17 MN is assumed for the boring force.

As the boring force is constant, the total required thrust force depends only on the friction force or, equivalently, the average shield pressure $\overline{\sigma }_{R}$ (Eq. 6). In a discrete simulation, $\overline{\sigma }_{R}$ depends in general on the in-situ stress σ_0_, on the material constants of the ground (*E, ν, f*_*c*_*, ϕ, ψ, η*), on the shield and lining stiffnesses (*K*_*s*_*, K*_*l*_), on the geometric parameters (*R, L,* Δ*R, L*_*T*_) and on the process parameters (v_*N*_*, N,* Δ*T*_1_*,* Δ*T*_2_*,* Δ*T*_3_*,* Δ*T*). Considering the parameter dimensions, the inverse proportionality between the Young’s modulus *E* and the displacements in elastoplastic media (Anagnostou and Kovári 1993; Ramoni and Anagnostou 2010b) and the interdependencies of some parameters (e.g., Δ*T*_1_ = *L*_*T*_ / v_*N*_), $\overline{\sigma }_{R}$ can be expressed in the following non-dimensional form:

9 $$\frac{{\overline{\sigma }_{R} }}{{\sigma_{0} }} = f\left( {\frac{E\Delta R}{{\sigma_{0} R}},\nu ,\;\frac{{f_{c} }}{{\sigma_{0} }},\phi ,\;\psi ,\frac{L}{R},\;\frac{{K_{s} R}}{E},\frac{{K_{l} R}}{E},\frac{{\eta {\text{v}}_{N} }}{ER},\frac{{L_{T} }}{R},\frac{{\Delta T_{2} }}{{\Delta T_{1} }},\frac{{\Delta T_{3} }}{\Delta T},\frac{{{\text{v}}_{N} \Delta T}}{R}} \right).$$

In the proposed semi-discrete simulation, the list of parameters reduces as follows:

10 $$\frac{{\overline{\sigma }_{R} }}{{\sigma_{0} }} = f\left( {\frac{E\Delta R}{{\sigma_{0} R}},\nu ,\;\frac{{f_{c} }}{{\sigma_{0} }},\phi ,\;\psi ,\frac{L}{R},\;\frac{{K_{s} R}}{E},\frac{{K_{l} R}}{E},\frac{{\eta {\text{v}}_{S} }}{ER},\frac{{\Delta T_{3} }}{\Delta T},\frac{{{\text{v}}_{S} \Delta T}}{R}} \right),$$

and in the fully continuous simulation as follows:

11 $$\frac{{\overline{\sigma }_{R} }}{{\sigma_{0} }} = f\left( {\frac{E\Delta R}{{\sigma_{0} R}},\nu ,\;\frac{{f_{c} }}{{\sigma_{0} }},\phi ,\;\psi ,\frac{L}{R},\;\frac{{K_{s} R}}{E},\frac{{K_{l} R}}{E},\frac{{\eta {\text{v}}_{G} }}{ER}} \right).$$

Using Eqs. 7 and 8, the dimensionless arguments of Eqs. 10 and 11 related to time-dependency can be expressed as functions of the independent dimensionless parameters of Eq. 9.

Section 5.1 evaluates the accuracy of the semi-discrete simulation of the stop-and-go phase, while Sect. 5.2 considers a full operation cycle, investigating the adequacy of the continuous and semi-discrete simulations. In both sections the basic behaviour is analysed first, followed by a parametric study that considers a common range of normalised net advance rates *η* v_*N*_* /E/R* = 10^–5^–10^4^ and a constant normalised segment length *L*_*T*_/*R* = 0.2.

### Stop-and-go Phase

**Fig. 10:**
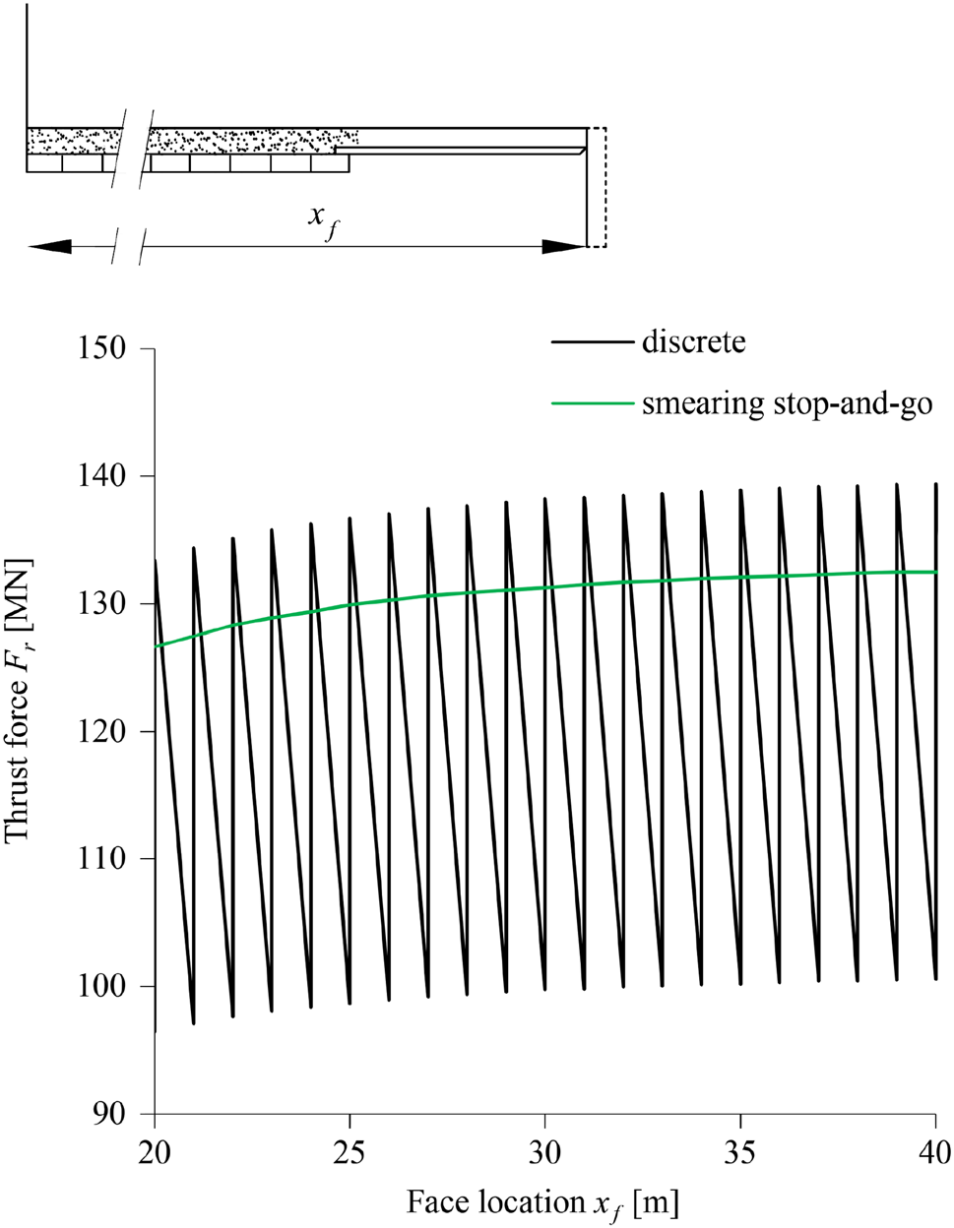
Thrust force evolution during the stop-and-go TBM operation based on discrete and semi-discrete simulations (Δ*R* = 0.25 m, *L*_*T*_ = 1 m, v_*N* _= 50 m/d, v_*s* _= 25 m/d,* η* = 60 MPa·d, Δ*T*_2_ = Δ*T*_1_; other parameters: Table 2)

The effect of the proposed smearing of a stop-and-go advance (Eq. 8) is examined in Fig. 10, which shows the typical development of the thrust force along the tunnel according to the results of a discrete simulation (TBM strokes with continuous advance for Δ*T*_1_ with v_*N*_, alternating with standstills for Δ*T*_2_) and a semi-discrete simulation (continuous advance with v_*S*_ and no standstill). The discrete simulation predicts a saw-shaped curve where the upper-values correspond to the TBM restart phases and the low-values correspond to the continuous advance phases. The smeared simulation, by contrast, predicts a smooth curve, which captures reasonably well the upper values of the discrete simulation.

**Fig. 11:**
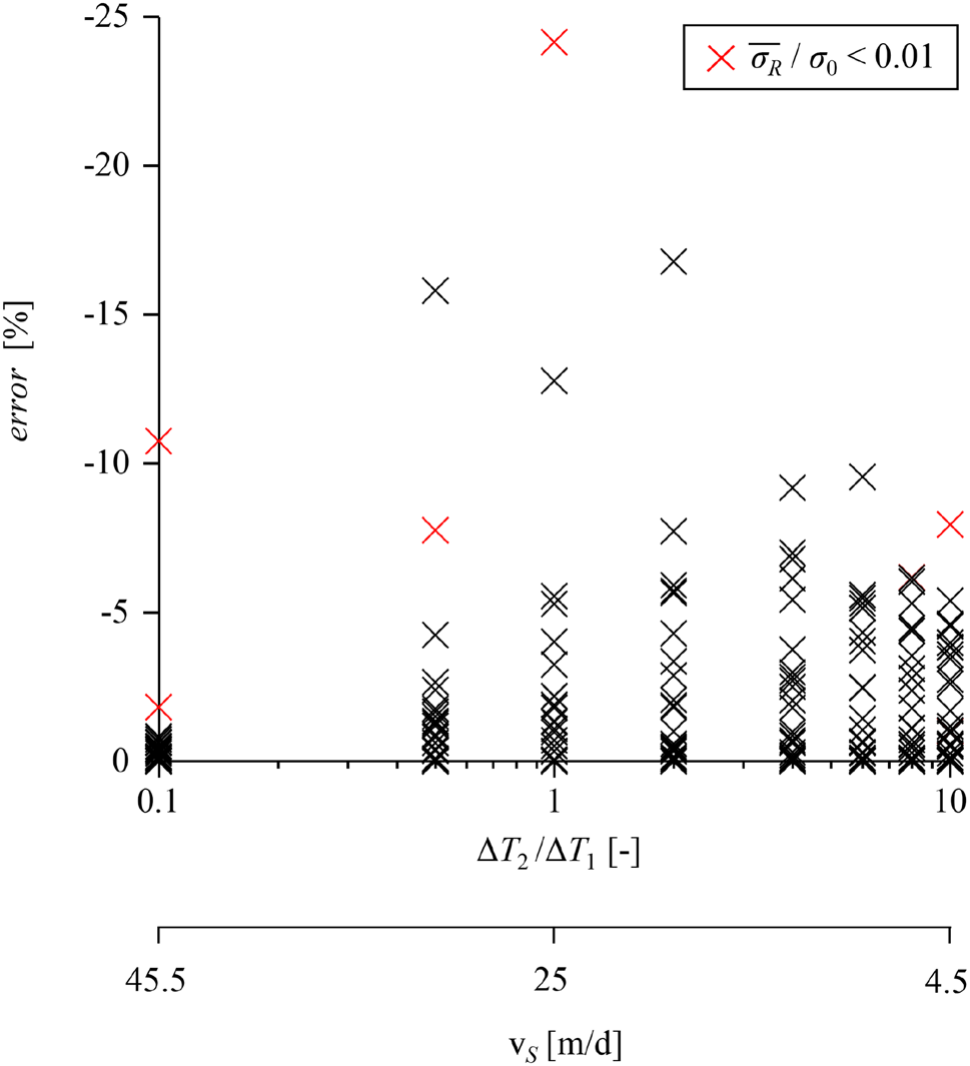
Error of semi-discrete simulations of the stop-and-go phase with respect to discrete simulations versus normalised lining erection duration (*E*Δ*R*/σ_0_/*R* = 1, 2; *f*_*c*_/*σ*_0 _= 0.05, 0.2; *ϕ* = 25°; *L*/*R* = 2;* K*_*s*_*R/E* = 10*; K*_*l*_*R/E* = 0.5*; η*v_*N*_*/E/R* = 10^–5^, 10^–4^, …, 10.^4^; *L*_*T*_/*R* = 0.2; Δ*T*_3_/Δ*T* = 0, other parameters: Table 2; 640 computations in total)

Figure 11 shows the error of semi-discrete simulations (with respect to fully discrete simulations) as a function of the ratio Δ*T*_2_* /*Δ*T*_1_. As expected, the error is smallest for very small Δ*T*_2_* /*Δ*T*_1_, where the advance becomes practically continuous. In all other cases, except for 5 outliers, the overall error falls below 10%, indicating that the accuracy of a semi-discrete approach over the stop-and-go phase is adequate in engineering terms.

### Full Excavation Cycle

**Fig. 12:**
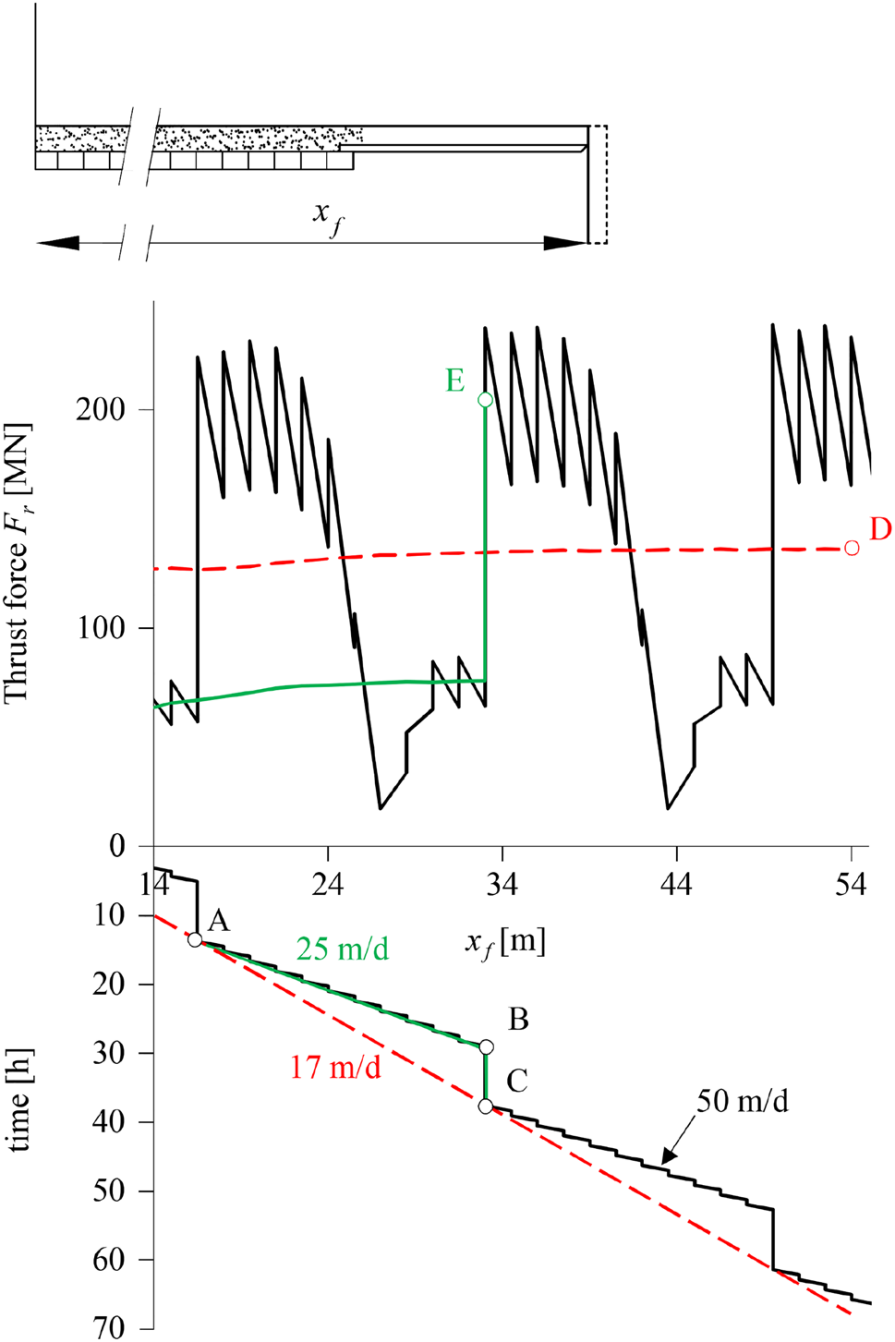
Thrust force evolution during two full excavation cycles based on discrete, semi-discrete and continuous simulations (Δ*R* = 0.25 m, *L* = 10.5 m, *L*_*T*_ = 1.5 m, *η* = 100 MPa·d, Δ*T*_2_ = Δ*T*_1_ = 0.72 h, Δ*T*_3_ = 8 h, Δ*T* = 24 h, v_*N* _= 50 m/d, *v*_*s* _= 25 m/d, v_*G* _= 17 m/d; other parameters: Table 2)

Full excavation cycles consisting of 16 h long stop-and-go TBM operations alternating with 8 h long maintenance standstills (2 + 1 shifts) are considered. Figure 12 shows the time-face location diagram (bottom) and the required thrust force along the tunnel (top), as determined from the three simulations, that is from the discrete simulation (black lines; v_*N*_ = 50 m/d), the semi-discrete simulation (green lines; v_*S*_ = 25 m/d) and the continuous simulation (red lines; v_*G*_ = 17 m/d).

During the 8 h maintenance standstill at *x*_*f*_ = 16.5 m (point A) the necessary thrust force increases by a factor of 4 (from 56 to 224 MN) because the shield partially prevents the development of the time-dependent deformations. Upon restart of the TBM (point A), the thrust force remains high during the advance through the plastified ground ahead of the tunnel face, starts decreasing as the TBM approaches the plastic zone boundary, and rapidly drops (complete unloading) when the shield exits the plastic zone and enters the adjacent, still elastic, rock. Subsequently, it starts increasing again due to ground plastification ahead of the face, reaching lower values in comparison to point A (*ca.* 56 MN, similar to the stop-and-go phase preceding point A). After the stop-and-go period (point B), the thrust force increases again substantially during the maintenance standstill (*ca.* 240 MN; point C), and the whole excavation cycle is repeated thereafter. The continuous simulation (red line) underestimates the maximum thrust force by more than 40% (point D: 140 vs. 240 MN), while the semi-discrete simulation (green line) approximates it to within 15% (point E: 205 vs. 240 MN).

**Fig. 13:**
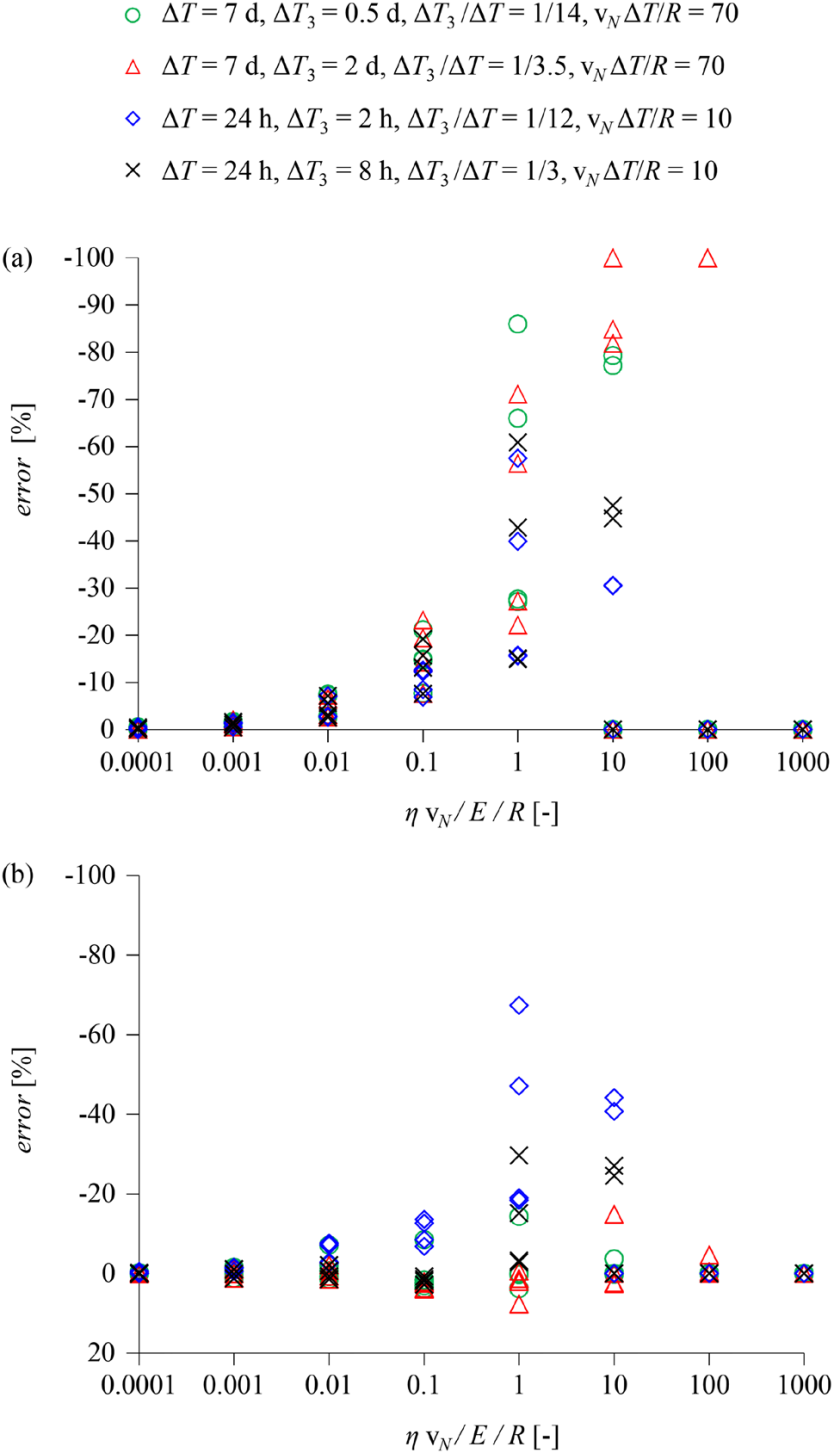
Error of continuous (**a**) and of semi-discrete simulations (**b**) of a full excavation cycle with respect to discrete simulations versus normalised net advance rate *η *v_*N*_* /E/R* in four stop-and-go and standstill scenarios (*E*Δ*R*/σ_0_/*R* = 1, 2; *f*_*c*_/σ_0_ = 0.05, 0.2; *ϕ* = 25°; *L*/*R* = 2; *K*_*s*_*R*/*E* = 10, *K*_*l*_*R*/*E* = 0.5; *L*_*T*_/*R* = 0.2; Δ*T*_2_/Δ*T*_1_ = 1, other parameters: Table 2; 320 computations in total for each diagram)

Figure 13a and b show the error of continuous and semi-discrete simulations, respectively, with respect to fully discrete simulations, as a function of the normalised net advance rate *η *v_N_ /*E*/*R*. The parametric study assumes Δ*Τ*_2 _/Δ*Τ*_1_ = 1 (*i.e*., the time for one lining ring erection equals to the duration of one TBM stroke), Δ*Τ*_3 _/ΔΤ = 1/14, 1/12, 1/3.5, 1/3, and v_*N *_Δ*Τ*/*R* = 10, 70, which for a 10 m diameter tunnel and a net advance rate of 50 m/d covers the following realistic scenarios:

- 6.5 d stop-and-go followed by a 0.5 d standstill (Δ*Τ*_3_ = 0.5 d, Δ*T* = 7 d);
- 5 d stop-and-go followed by a 2 d standstill (Δ*Τ*_3_ = 2 d, Δ*T* = 7 d);
- 22 h stop-and-go followed by a 2 h standstill (Δ*Τ*_3_ = 0.083 d, Δ*T* = 1 d);
- 16 h stop-and-go followed by an 8 h standstill (Δ*Τ*_3_ = 0.33 d, Δ*T* = 1 d).

For very small viscosities or advance rates, the error is zero, as expected, since there is no time-dependency. The error increases with increasing viscosity in both cases, exceeding 30% in continuous simulations for values *η *v_*N*_/*E*/*R* greater than 1. (The 100% errors for very high *η* v_*N*_/*E*/*R* correspond to cases where the continuous simulation predicts zero shield pressure.) The error of the semi-discrete simulation is considerably smaller and does not exceed 20% for *η *v_*N*_/*E*/*R* below 0.1–1 or beyond 10. In the range 1–10 the error is considerable, indicating that the proposed approach is inapplicable and that TBM simulations must consider even the short standstills of the stop-and-go process explicitly.

## Application Examples

The importance of considering the effects of creep (Sect. 4) and adequately modelling the excavation standstills during the construction process (Sect. 5) is demonstrated in this section via two application examples. The first example analyses the TBM drive during construction of the Fréjus safety gallery, whereas the second presents a feasibility assessment of a TBM drive through the conventionally excavated Sedrun North critical zone of the Gotthard base tunnel.

### Fréjus Safety Gallery

The 13 km long Fréjus highway tunnel connecting Modane (France) to Bardonecchia (Italy) is located on the French-Italian border in the Western Alps. Construction started in 1974 by conventional tunnelling (full-section drill and blast with 4.5 m advance steps) and the tunnel opened to road traffic in 1980. The single main tube of the tunnel has an approximately NS orientation and crosses three alpine lithotypes at a maximum depth of 1800 m, the principal being the Piemontaise zone that consists of calc-schist with phyllitic and carbonate facies. In 2009 works started on a parallel safety gallery located 50 m from the highway tunnel. The 6.5 km long stretch of the gallery in France was excavated between 2011 and 2013, with drill and blast over the first 650 m and with a single shield TBM over its remaining part. The latter crosses black and green schist between chainages 650–1500 m, anhydrite between chainages 1500–1750 m, and calc-shist between chainages 1750–6500 m (Schivre et al. 2014; De la Fuente et al. 2020).

To ensure that the TBM was able to adjust to various adverse scenarios previously encountered during the highway tunnel construction in the 70s, the following specifications were used (Schivre et al. 2014): shield length of 11.2 m; nominal shield diameter of 9.37 m with conicity of 60 mm on the diameter; nominal boring diameter of 9.46 m (90 mm overcut on the diameter at the tunnel crown in the shield front), adjustable to 9.56 m or 9.66 m (to accommodate an increase of the overcut by 100 and 200 mm on the diameter, respectively); and installed thrust force of 106 MN.

Monitoring data were collected during the TBM drive from 10 hydraulic jacks installed over the upper shield extrados. The data indicated maximum average convergences of up to 300 mm in two zones in calc-schist, between chainages 1750–3000 m and 4300–6500 m (*cf.* Fig. 8 in Vinnac et al. 2014). The higher convergences in these zones are partially attributed to buckling of rock layers along the schistosity planes oriented parallel to the tunnel axis, which resulted in the detachment of rock blocks and their collapse on the shield and lining. Despite these effects, the TBM specifications, the use of the intermediate overcut in parts and the continuous adjustment of the TBM operation based on real-time monitoring data, enabled an uneventful drive with a moderate thrust force up to 30 MN in the calc-shist zone. The maximum thrust force of 52 MN was recorded in the anhydrite zone (*ca*. chainage 1550 m), where the recorded convergences were, however, much lower (*cf.* Fig. 8 in Vinnac et al. 2014).

The present application example analyses the TBM drive through the calc-schist zone, based upon fully discrete transient numerical simulations that consider the effects of creep (Sect. 3). Comparative computations are also conducted to assess the accuracy of the time-independent (*η* = 0) and time-dependent (*η* > 0) continuous models usually employed, and of the semi-discrete model proposed in Sect. 5. The simulations do not consider the anhydrite zone, where the maximum thrust force was recorded, since the adopted computational model is not suitable for capturing the additional time-dependency related to chemical processes in anhydrite (“swelling”).

#### Adopted Parameters

The parameters adopted in the simulations are given in Table 3. A *t*_95_ = 60–120 days, indicating moderate creep intensity, is estimated from monitoring data for the convergence evolution over time at chainage 5080 m of the highway tunnel. The data concerns the most unfavourable direction over the horseshoe-shaped profile, normal to the schistosity planes (*cf.* Fig. 4b in De la Fuente et al. 2020), and was recorded prior to the installation of the final lining. The estimated *t*_95_ is used to determine the expected range of viscosity *η* after Fig. 1a (open tunnel profile with low support resistance), considering additionally two parameter sets for calc-schist: (i) the set reported by Vinnac et al. (2014), and, (ii), a set calibrated based on the monitoring data. For simplicity, set (ii) is chosen to be identical to (i), except for the Young’s modulus *E*; the latter is adjusted such that the maximum radial displacement predicted by a ground response curve (GRC; Anagnostou and Kovári 1993) for an unsupported opening matches the one of the monitoring data. The GRC for set (i) gives a maximum displacement of *ca.* 600 mm on the diameter, which includes the ground pre-deformation ahead of the tunnel face. On the other hand, the monitoring data indicates maximum long-term convergence (*i.e.,* displacement relative to the tunnel face) of *ca.* 480 mm on the diameter, in the most unfavourable cross-sectional orientation. Assuming that the pre-deformation is 50% of the total displacement far behind the face (typically 30–70%), the latter is *ca.* 2*480 = 960 mm. A Young’s modulus of 7500*600/960 = *ca*. 4700 MPa is thus adopted in set (ii), 1.6 times lower compared to set (i). Since *f*_*c*_ and *ϕ* are identical in the two sets, *η* is estimated at the same point of Fig. 1a using only different *E* values; the *η*-ranges of the two sets are thus linearly dependent by a factor of 1.6.

**Table 3:** Parameters considered in application examples

			Fréjus safety gallery	Gotthard base tunnel
Ground				
Young’s modulus	*E*	[MPa]	7500^a^, 4700^b^	1000 ± 15%^r^
Poisson’s ratio	*ν*	[–]	0.2^a^	0.25
Angle of internal friction	*ϕ*	[°]	35^a^	27 ± 3^s^
Dilatancy angle	*ψ*	[°]	15^c^	5 ± 2^s^
Uniaxial compressive strength	*f*_*c*_	[MPa]	3.8^a^	1.3 ± 0.3^s^
Time to reach 95% of displacement	*t*_95_	[d]	60–120^d^	
Viscosity	*η*	[MPa^.^d]	2700–5400^e^ 1700–3400^f^	10–100^t^
Overburden	*H*	[m]	1200–1400^g^	800
In-situ stress	*σ*_0_	[MPa]	40^h^	20
TBM				
Nominal boring radius	*R*	[m]	4.73^i^	5
Shield Length	*L*	[m]	11.2^i^	10
Overcut	Δ*R*	[mm]	60, 110^j^	200
Shield thickness	*d*_*s*_	[mm]	75	75
Young’s modulus of the shield (steel)	*E*_*s*_	[GPa]	210	210
Shield stiffness^k^	*K*_*s*_	[MPa/m]	704	630
Coefficient of shield skin friction	*µ*	[–]	0.3, 0.4^l^	0.1, 0.15^u^
Maximal cutter force^m^	*F*_*c*_	[kN]	267	267
Number of cutters	*n*	[–]	63^i^	63^v^
Thrust force (boring process)^n^	*F*_*b*_	[MN]	17	17
Lining				
Lining thickness	*d*_*l*_	[mm]	400^i^	500
Young’s modulus of the lining (concrete)	*E*_*c*_	[GPa]	35	35
Lining stiffness^k^	*K*_*l*_	[MPa/m]	626	700
Segmental ring width	*L*_*T*_	[m]	1.8^i^	1.5
Construction process				
Time for one ring advance^o^	Δ*T*_1_	[min]	28	36
Time for one ring build	Δ*T*_2_	[min]	30	30
Duration of the longer standstill	Δ*T*_3_	[h]	8	8
Time for one cycle	Δ*T*	[h]	24	24
Net advance rate	*v*_*N*_	[m/d]	94^p^	60
Smeared stop-and-go advance rate^q^	*v*_*S*_	[m/d]	45	33

^a^Residual values after Table 1 in Vinnac et al. (2014)

^b^Calibrated based on monitoring data at Chainage 5080 of the Fréjus road tunnel (De la Fuente et al. 2020); see Sect. 6.1

^c^*ψ* = *ϕ*–20°

^d^Estimated from monitoring data of convergence development over time at Chainage 5080 (Fig. 4b in De la Fuente et al. 2020)

^e^Estimated after Fig. 1a for *f*_*c*_/*σ*_0_ = 0.1, *ϕ* = 35°, *t*_95_* E*/*η* = 167, *t*_95_ = 60–120 d, *E* = 7500 MPa

^f^Estimated after Fig. 1a for *f*_*c*_/*σ*_0_ = 0.1, *ϕ* = 35°, *t*_95_* E*/*η* = 167, *t*_95_ = 60–120 d, *E* = 4687.5 MPa

^g^After Fig. 8 in Vinnac et al. (2014) for the critical squeezing zone between chainages 4500 and 5300 m

^h^Rounded value of higher horizontal in-situ stress (lateral earth pressure coefficient of 1.2–1.4), considering overburdens of 1200–1400 m and unit weight of 27 kN/m^3^ (Vinnac et al. 2014)

^i^Schivre et al. (2014)

^j^Equivalent uniform overcuts for rotationally symmetric analyses; taken equal to the value in the middle of the shield length, considering nominal and intermediate overcuts of 90 and 190 mm on the diameter in the shield front, and conicity of 60 mm on the diameter after Schivre et al. (2014): (90 + 60/2)/2 = 60 mm, (190 + 60/2)/2 = 110 mm on the radius

^k^*K*_*l*_ = *E*_*l*_* d*_*l*_*/R*^*2*^; *K*_*s*_ = *E*_*s*_* d*_*s*_*/R*^*2*^

^l^Sliding and static friction coefficient without shield extrados lubrication; after Table 2.3 in Ramoni and Anagnostou (2011a)

^m^AfterSänger (2006)

^n^*F*_*b*_ = *n F*_*c*_

^o^ΔT_*1*_ = *L*_*T*_ /v_*N*_

^p^Average of 50–80 mm/min after Fig. 8 in Vinnac et al. (2014)

^q^Eq. (6)

^r^After Fig. 5 in Vrakas et al. (2018), *E*_50_-value for 20 MPa in–situ stress

^s^After Table 1 in Vrakas et al. (2018)

^t^After Sect. 5.5.1 in Cantieni et al. (2011)

^u^Sliding and static friction coefficient with shield extrados lubrication after Table 2.3 in Ramoni and Anagnostou (2011a)

^v^Assumed value; taken equal to that of the Fréjus application example

#### Analysis of TBM Drive

**Fig. 14:**
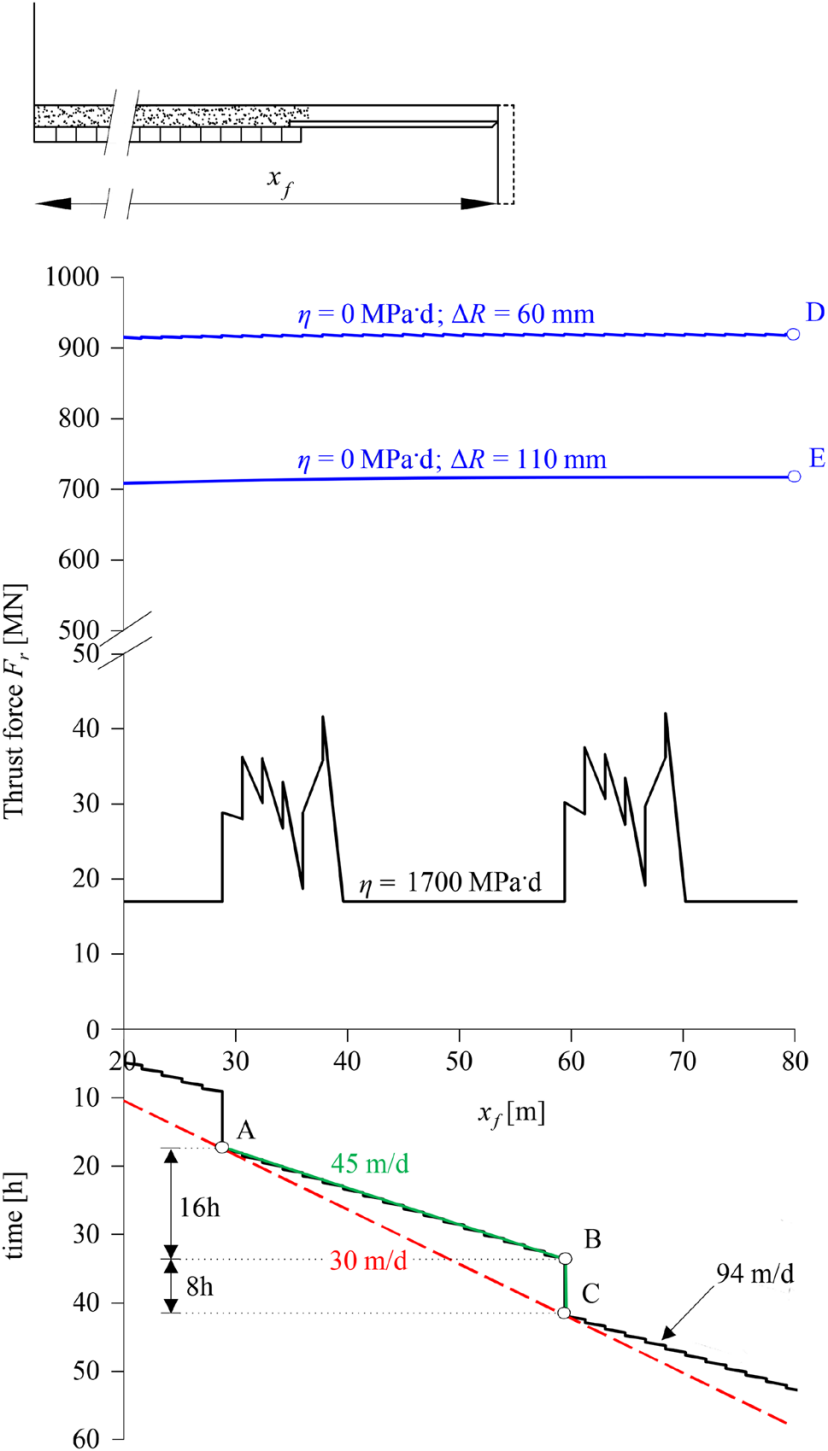
Fréjus safety gallery: Thrust force evolution predicted by discrete and time-independent continuous simulations of two full excavation cycles (parameters: Table 3)

The fully discrete model predicts contact between shield and ground only when considering the nominal overcut of 60 mm and the lowest viscosity value of 1700 MPa^.^d for set (ii). Figure 14 shows with black lines the time-face location diagram (bottom) and the required thrust force along the tunnel (top) for this case. Analogously to Sect. 5 two full excavation cycles are considered, each consisting of 16-h long stop-and-go TBM operations (2 shifts; part between points A, B) alternating with 8 h long maintenance phases (1 shift; part between points B, C), with realistic typical durations for the various operations (Table 3). The maximum predicted thrust force is 41 MN, very close to the maximum value of 30 MN recorded in the critical zones (Fig. 8 in Vinnac et al. 2014). Differences may be partially attributed to the asymmetric loading of the shield resulting from the detachment of blocks following buckling, which cannot be captured by the adopted rotationally symmetric computational model (Sect. 3). When considering the nominal overcut with higher viscosity values for any of the two parameter sets, or the intermediate overcut of 110 mm with any of the viscosity values and parameter sets, the model predicts no contact between shield and ground, and hence the thrust force is constant and equal to the boring force (these cases are not shown in Fig. 14).

#### Accuracy Evaluation of Semi-discrete and Continuous Simulations

Both the semi-discrete and the continuous time-dependent models fail to capture the shield ground contact that actually occurred, for both overcuts and viscosity ranges. The prediction error is moderate and justifiable in this specific case, due to the relatively high viscosity values and the very conservative TBM specifications. The accuracy of these models can thus not be assessed in this example. Either way, the applicability of the semi-discrete model would be questionable in this case, since the normalised net advance rate *η *v_*N*_ /*E*/*R* ranges between 7 and 14, which overlaps with the region of highest errors in Fig. 13b.

Most interesting are the results of the time-independent continuous models. These overestimate the required thrust force excessively, predicting values between 720 MN for a 110 mm overcut (point E in Fig. 14) and 920 MN for a 60 mm overcut (Point D in Fig. 14), *ca.* 24–30 times higher compared to those observed in reality. Such values lie far beyond technical limits for TBMs and would be thoroughly misleading for a feasibility assessment of the TBM drive. This result clearly demonstrates that relying on continuous time-independent models in cases of moderately to pronouncedly creeping ground may prove excessively conservative, erroneous and misleading.

### Gotthard Base Tunnel

The 57 km long Gotthard Base railway tunnel in Switzerland was constructed between 1999 and 2011 and opened to traffic in 2016. Its two main tubes cross four geological zones from north to south: the Aar Massif (AM), the Intermediate Tavetsch Massif (TM), the Gotthard Massif (GM) and the penninic gneiss (PM) (Mezger et al. 2013; Vogelhuber et al. 2023). Over the major part of the tunnel crossing the AM, GM and PM zones, which consist of competent gneisses and granites with very few local geologically disturbed regions, the conditions for tunnelling were favourable overall and hard rock TBMs were used. Conversely, over the 1100 m long stretch crossing the southern part of the AM zone (Clavaniev zone) and the northern part of the TM zone at 800 m depth, heavily and pronouncedly variable squeezing conditions were both anticipated and encountered during construction. This critical zone consists of alternating layers of kakiritic gneisses, slates and phyllites, and is commonly referred to as “Sedrun North”. The observed variability of squeezing is attributed to differences in the degree of kakiritization, the schistosity orientation and the competent-weak rock layer alternations (Mezger et al. 2013).

The unfavourable conditions for mechanised tunnelling in Sedrun North necessitated resorting to conventional, full-face excavation of the 10 m diameter circular tunnel cross-section, with over-excavation of up to 0.8 m, systematic anchoring of the cross-section and various auxiliary measures, including systematic bolting of the tunnel face (Vogelhuber et al. 2023). In the first stage, a yielding support was installed, consisting of two overlain sliding steel rings connected by friction loops, to accommodate convergences in a controlled manner and allow for stress relief of the ground. In the second stage, after the rate of convergences slowed down, a 0.3–0.6 m thick shotcrete ring was initially applied, followed later by an in situ-cast final lining with maximum thickness of 1.2 m. The maximum average radial convergence was *ca.* 40 cm, with peak values as high as 70 cm measured at specific points over the tunnel boundary (Kovári and Ehrbar 2008).

The main aim of the present example is to reassess the feasibility of the TBM drive through Sedrun North, considering our knowledge today about the mechanical behaviour of the kakirites, the latest thrust prediction methods and the current state of TBM technology. Concerning the latter, Ramond and Schivre (2019; pp. 32) provide an overview of the installed nominal thrust in single-shield TBMs used in some of the most important tunnelling projects between 2000 and 2019. One can readily identify values as high as, e.g., 140 MN in the Pajares tunnel in Spain, or 155 MN in the Brenner Base tunnel crossing Austria and Italy. Installing additional thrust is possible with an appropriate modification of the TBM, e.g., by installing removable auxiliary hydraulic jacks. This approach was used during the construction of section 4 of the 10 m diameter Pajares tunnel between 2007 and 2009, where the nominal thrust of 140 MN was initially increased to 193 MN and subsequently to 225 MN, following jamming of the TBM due to the pronounced squeezing conditions (Ramoni and Anagnostou 2010b). Considering that an installed thrust of 225 MN was materialised 15 years ago, moderately higher values appear thoroughly feasible with incremental developments of current technology.

The feasibility of mechanised tunnelling is assessed with the aid of fully discrete, transient numerical simulations which consider the effects of creep and a reliable set of parameters collected from various rigorous investigations on the Gotthard Base tunnel. Additionally, comparative computations are performed to assess the adequacy for decision-making during design of the proposed semi-discrete model (Sect. 5), the time-independent (*η* = 0) and time-dependent (*η* > 0) continuous models (which currently constitute the standard in both engineering practice and research), and the design nomograms of Ramoni and Anagnostou (2010b).

#### Adopted Parameters

The parameters adopted in the computations are given in Table 3 and discussed hereafter.

The elasticity and plasticity parameter ranges are taken after Vrakas et al. (2018), which consider the results of 90 consolidated drained triaxial compression tests on kakiritic samples from Sedrun North. During the project planning phase between 1998 and 2000, 55 of these tests were conducted (Vogelhuber 2007), and a further 35 during the construction phase between 2004 and 2007 (Anagnostou et al. 2008), as part of a large experimental programme undertaken by the ETH Rock Mechanics laboratory.

The range of viscosity *η* = 10–100 MPa^.^d corresponds to the one estimated by Cantieni et al. (2011), based on in-situ monitoring data for the tunnel face extrusion during a construction standstill at chainage 2090 m. The data was recorded using reverse-head-extensometers installed along the tunnel axis and showed that the extrusion stopped after 30 days and that 95% of its final value was achieved within about 20 days, thereby indicating a rather low viscosity.

A single shield TBM with shield length equal to the 10 m tunnel diameter is considered. Very low sliding and static shield skin friction coefficients are adopted (*μ* = 0.1 and 0.15, respectively), which can be achieved via lubrication of the shield extrados (*cf.* Table 2.3 in Ramoni and Anagnostou 2011a), and a large overcut is assumed (Δ*R* = 200 mm), which can be materialized with existing overboring systems (*cf.* Table 2.2 in Ramoni and Anagnostou 2011a). These specifications are deliberately selected to be as favourable as possible, while typical specifications for the shield and lining are otherwise considered. Analogously to Sect. 5, two full excavation cycles are assumed, consisting of 16-h stop-and-go TBM operations (2 shifts) alternating with 8-h maintenance phases (1 shift) with typical, realistic durations.

#### Feasibility Assessment

**Fig. 15:**
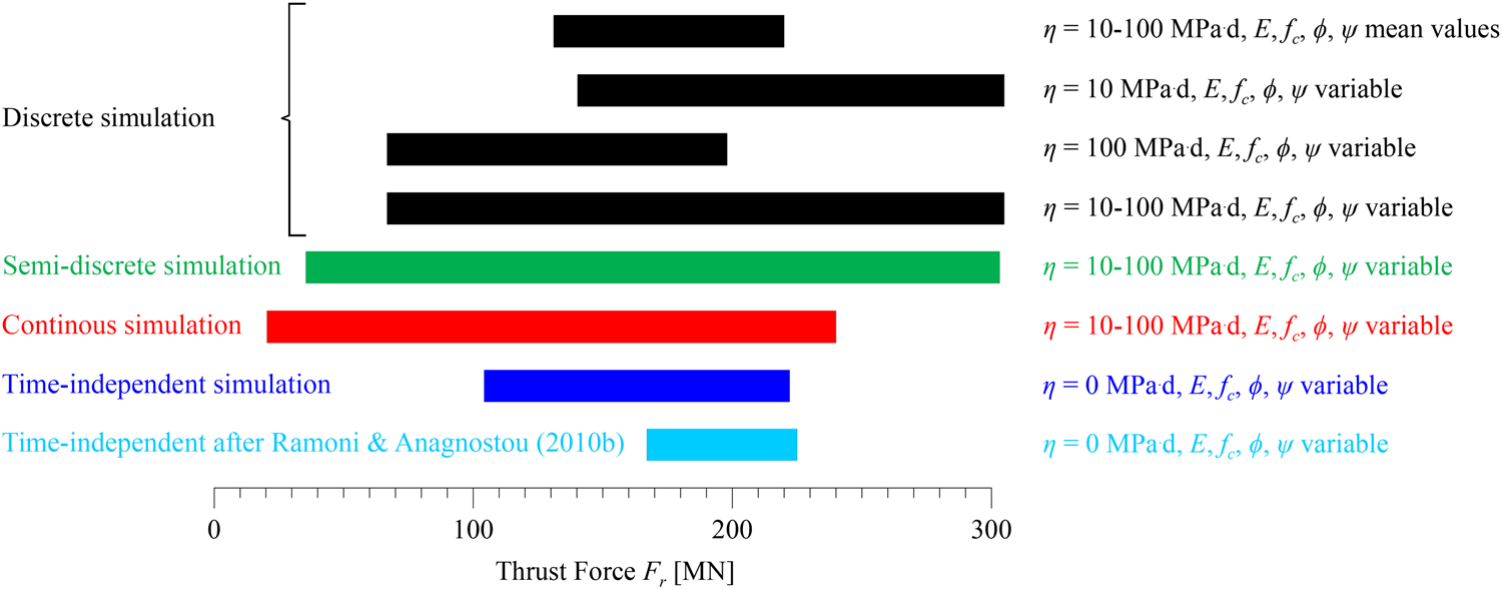
Gotthard base tunnel: Predicted thrust force range from discrete, semi-discrete and continuous time-dependent and time-independent simulations of two full excavation cycles (parameters: Table 3)

Figure 15 shows the predicted range of the required thrust force, considering the inherent uncertainties associated with the stiffness, strength, and viscosity of the ground (*cf.* Table 3), as well as different simulation methods. The feasibility assessment is discussed with reference to the four black bars, which correspond to fully discrete simulations. From top to bottom, these show the predicted range considering: (i) the expected range of viscosity *η* = 10–100 MPa^.^d in combination with the mean strength and stiffness parameters; (ii) the minimum expected viscosity *η* = 10 MPa^.^d in combination with the expected range of the strength and stiffness parameters; (iii) the maximum expected viscosity *η* = 100 MPa^.^d in combination with the expected range of the strength and stiffness parameters; and, (iv), the expected ranges of the viscosity, strength and stiffness parameters.

A comparison of the 1st bar with the 2nd and 3rd bars shows that the uncertainty associated with the strength and stiffness parameters of the ground is far more critical than that associated with the viscosity and leads to a much broader range. The 4th bar, which embeds all uncertainties, indicates a maximum thrust force of 300 MN, which corresponds to the combination of the minimum viscosity *η* = 10 MPa^.^d with the minimum strength and stiffness (upper end of bar 2).

It must be considered that the determined value of 300 MN corresponds to the worst possible combination of all material parameters, which is also assumed to prevail along the entire length and over the entire cross-section of the tunnel (due to the assumptions of homogeneous ground and rotational symmetry, respectively; *cf.* Section 3). In reality, such a combination is very unlikely and could only occur over narrow weak zones interspersed between zones of more or less competent rock with superior stiffness and strength properties. The presence of competent zones is a well-established characteristic of the geological formations in Sedrun North (*cf.* Mezger et al. 2013), and has a favourable influence with respect to shield jamming: it reduces ground deformations in the weaker zones due to the shear stresses mobilised at their interfaces and the resulting longitudinal arching effect of the ground (so-called “wall-effect”). The value of 300 MN is thus a clearly pessimistic estimate, and lower values would be more realistic.

However, based upon the preceding discussion regarding feasible thrust force values in single shield TBMs, even an installed thrust of 300 MN appears to be a viable prospect with current technological developments. An increase of 75 MN with respect to the 225 MN that was materialised 15 years ago is not extreme and can probably be accommodated with a suitable TBM design. In conclusion, from today’s perspective, a TBM drive prospect appears feasible overall.

#### Accuracy Evaluation of Semi-discrete and Continuous Simulations and Design Nomograms

In the following, the adequacy of the proposed semi-discrete, continuous time-dependent and time-independent models, and of the design nomograms of Ramoni and Anagnostou (2010b) is assessed with reference to the results of the fully discrete model. The comparison is based on the respective predicted ranges of thrust force shown in Fig. 15, considering the mechanical parameters and viscosity of the ground as variable within their expected ranges.

The semi-discrete model (green bar) captures very accurately the range of the fully discrete one (4th black bar), predicting an almost identical maximum value and a slightly lower minimum value (which is anyway irrelevant from a feasibility assessment viewpoint). Therefore, it allows for the same conclusions to be drawn in practical engineering terms also within the context of the feasibility assessment discussed previously.

Conversely, the continuous time-dependent model (red bar) systematically underestimates the predictions of the fully discrete one (4th black bar). While the underestimation of the low-end of the range is subcritical, the underestimation of the high-end is misleading for feasibility assessment and decision-making. Specifically, the 240 MN maximum value is sufficiently high to raise an alert, but otherwise points towards a thoroughly feasible TBM drive, especially when the favourable influence of the wall-effect is considered.

The same applies to the continuous time-independent model (blue bar), which predicts an even lower required thrust force of 220 MN that is thoroughly feasible even without considering the wall-effect influence. The fact that the time-independent model prediction is lower than that of time-dependent simulations is attributed to the paradox discussed in Sect. 4 and shown in Fig. 6, which manifests itself within the expected range of viscosity *η* = 10–100 MPa^.^d.

The design nomograms (light blue bars) of Ramoni and Anagnostou (2010b), which were established on the basis of the time-independent continuous simulations, predict the same maximum value, as expected. The only difference is that the nomograms embed the conservative assumptions of very high and very low stiffnesses for the shield and lining, respectively, and hence they systematically overestimate the low end of the range predicted by continuous time-independent simulations that consider the actual stiffnesses.

## Conclusions

This paper presented an investigation into the effect of the time-dependency of squeezing on shield jamming and lining overstressing, along with a comparative evaluation of different approaches for simulating TBM advance and considering excavation standstills in transient numerical computations. The simplest possible MC-Perzyna constitutive model was adopted, which considers time-dependency solely in the plastic regime via a single viscosity parameter *η*, and allows the magnitude of the viscosity in tunnelling boundary value problems to be directly quantified and the results to be qualitatively interpreted in a simple manner (Sect. 2). The key contributions of the present work are summarised hereafter.

First, the common notion that creep is thoroughly favourable for shield jamming during advance has been disputed. Within a certain viscosity range, a counter-intuitive behaviour occurs, where the thrust force increases with increasing viscosity, due to the interplay of two counter-acting effects (Sect. 4): (i) viscosity delays the closure of the annular gap, thus reducing the contact area between shield and ground, while, (ii), it also limits plastic deformations, thus limiting stress relief in the ground ahead of the tunnel face and increasing the pressure transferred to the shield after every excavation increment. Beyond this range, the thrust force starts decreasing with increasing viscosity, since contact between shield and ground happens sufficiently far from the tunnel face for the effect of face stiffening to have an influence. The aforementioned paradox also appears in standstills and at steady-state conditions, where the thrust force required also increases due to the manifestation of additional viscoplastic deformations over time. This increase makes creep thoroughly unfavourable during standstills, even in the range of viscosities where its influence may be favourable during advance.

Second, creep has been shown to be thoroughly unfavourable for the steady-state lining loads far behind the face (Sect. 4). Viscosity reduces the plastic deformations in the vicinity of the face, and hence greater plastic deformations develop over time, which are constrained by the lining, thus increasing the pressure acting upon it.

Third, a semi-discrete approach has been proposed to simulate the TBM advance, which can be idealised as stop-and-go phases alternating with regular standstills for maintenance works (Sect. 5). This smears only the short standstills during the stop-and-go phase, but otherwise explicitly simulates the regular ordinary standstills. The proposed approach approximates the required thrust force to within 10% if a maintenance standstill is not considered (or is very small in proportion to the stop-and-go phase) and within 20% otherwise, provided that the values of the normalised net advance rate *η* v_*N*_/*E*/*R* lie outside the range 1–10. Therefore, it provides the same basis for decision-making as the “exact” discrete model in practical tunnel engineering situations, and outperforms the fully continuous simulations usually considered in the literature (Sect. 6).

## Data Availability

Not applicable.

## Constitutive Model Formulation and Implementation in Abaqus^®^

This appendix presents the formulation and implementation of the adopted constitutive model as a user-defined material subroutine (UMAT) in Abaqus^®^ (Dassault Systèmes 2018). For the general 3D case, the effective stress and strain vectors are denoted as $\tilde {\mathbf{\upsigma}}$ = {*σ*_*xx*_* σ*_*yy*_* σ*_*zz*_* τ*_*xy*_* τ*_*yz*_* τ*_*zx*_} and $\tilde{\varepsilon }$ = {*ε*_*xx*_* ε*_*yy*_* ε*_*zz*_* γ*_*xy*_* γ*_*yz*_* γ*_*zx*_}, respectively, in the material local coordinate system (*x*,*y*,*z*), and as **σ = **{*σ*_1_* σ*_2_* σ*_3_ 0 0 0} and **ε** = {*ε*_1_* ε*_2_* ε*_3_ 0 0 0} in the principal coordinate system, where the ordering *σ*_1_ ≥ *σ*_2_ ≥ *σ*_3_ is assumed. The geomechanics sign convention is adopted (compressive stresses/strains positive). All quantities are functions of time, and their derivatives with respect to time, henceforth referred to as rates or increments, are denoted with an upper dot symbol “ ^.^ ”.

### Elastic Constitutive Relationship

The linear elastic constitutive relationship is expressed in terms of the stress rate ${\dot{\mathbf{\sigma }}}$ and the elastic strain rate ${\dot{\mathbf{\varepsilon }}}^{el}$ in the principal coordinate system as

12 $${\dot{\mathbf{\sigma }}} = {\mathbf{D}}^{el} {\dot{\mathbf{\varepsilon }}}_{{}}^{el} ,$$

where ${\mathbf{D}}^{el}$ is Hooke’s constant elastic stiffness matrix.

### Yield condition

The MC yield condition is expressed in the principal stress space as.

13 $$f_{1} \left( {{\varvec{\upsigma}}} \right) = \sigma_{1} - m\,\sigma_{3} - f_{c} = 0,$$

where *m* is the yield surface slope in the principal stress plane *σ*_1_-*σ*_3_ and *f*_*c*_ the uniaxial compressive strength, which are functions of the friction angle *ϕ* and the cohesion *c*:

14 $$m = \frac{1 + \sin \phi }{{1 - \sin \phi }},$$

15 $$f_{c} = \frac{2c\cos \phi }{{1 - \sin \phi }}.$$

The yield condition (Eq. 13) geometrically represents a plane of the pyramidal MC yield surface, where the ordering *σ*_1_ ≥ *σ*_2_ ≥ *σ*_3_ holds. Along its lines of intersection with the adjacent planes, the following yield condition must be considered additionally in the case of triaxial compression *σ*_1_ ≥ *σ*_2_ = *σ*_3_:

16 $$f_{2} \left( {\varvec{\sigma}} \right) = \sigma_{1} - m\,\sigma_{2} - f_{c} = 0,$$

and the following in the case of triaxial extension *σ*_1_ = *σ*_2_ ≥ *σ*_3_:

17 $$f_{3} \left( {{\varvec{\upsigma}}} \right) = \sigma_{2} - m\,\sigma_{3} - f_{c} = 0.$$

The point of intersection of all three planes (MC yield surface apex), where the stress state is purely tensile in cohesive geomaterials, is not considered herein, as it is not relevant for the investigated tunnel boundary value problems.

### Consistency Condition

The rate of change of the yield functions is given by the following expression:

18 $$\dot{f}_{i} \left( {\varvec{\sigma}} \right) = {\mathbf{n}}_{i}^{T} \,\dot{\varvec{\sigma }},$$

where** n**_*i*_** = **d*f*_*i*_/d**σ** is the normal vector to the yield surface *f*_*i*_. In classic plasticity theory the stress state upon yielding remains on the yield surface, and hence the rate in Eq. 18 must be zero to ensure that the stress increments ${\dot{\mathbf{\sigma }}}$ are tangent to the surface boundary. In the framework of overstress theory (Perzyna 1966), however, viscoplastic deformations occur for stress states lying on or outside the yield surface (*f*_*i*_ (**σ**) ≥ 0), which means that ${\dot{\mathbf{\sigma }}}$ is not strictly tangent but may also be oriented outwards from the yield surface, and, in turn,$\dot{f}$ in Eq. 18 is generally non-zero.

### Strain Decomposition and Viscoplastic Flow Rule

The total strain rate can be decomposed into elastic and viscoplastic counterparts:

19 $${\dot{\mathbf{\varepsilon }}} = {\dot{\mathbf{\varepsilon }}}^{el} + {\dot{\mathbf{\varepsilon }}}^{vp} ,$$

where the elastic counterpart ${\dot{\mathbf{\varepsilon }}}^{el}$ obeys Hooke’s constitutive relationship in Eq. 12, and the viscoplastic counterpart ${\dot{\mathbf{\varepsilon }}}^{vp}$ is obtained from the flow rule. A non-associated flow rule is considered, which adopts Koiter’s (1953) generalisation for plastic flow along an edge of the yield surface:

20 $${\dot{\mathbf{\varepsilon }}}^{vp} = \left\{ {\begin{array}{*{20}c} {\dot{\lambda }_{1}^{vp} {\mathbf{r}}_{1} \, } \\ {\dot{\lambda }_{1}^{vp} {\mathbf{r}}_{1} + \dot{\lambda }_{2}^{vp} {\mathbf{r}}_{2} } \\ {\dot{\lambda }_{1}^{vp} {\mathbf{r}}_{1} + \dot{\lambda }_{3}^{vp} {\mathbf{r}}_{3} } \\ \end{array} } \right.\begin{array}{*{20}c} , \\ , \\ , \\ \end{array} \begin{array}{*{20}c} { \, f_{1} \ge 0 \wedge f_{2} \le 0 \wedge f_{3} \le 0,} \\ { \, f_{1} \ge 0 \wedge f_{2} \ge 0 \wedge f_{3} \le 0,} \\ { \, f_{1} \ge 0 \wedge f_{2} \le 0 \wedge f_{3} \ge 0.} \\ \end{array}$$

In the above expression **r**_*i*_** = **d*g*_*i*_ /d**σ** are the normal vectors to the plastic potential surfaces *g*_*i*_ that correspond to the yield surfaces *f*_*i*_ (Eqs. 13, 16, 17):

21 $$g_{1} \left( {{\varvec{\upsigma}}} \right) = \sigma_{1} - \kappa \,\sigma_{3} ,$$

22 $$g_{2} \left( {{\varvec{\upsigma}}} \right) = \sigma_{1} - \kappa \,\sigma_{2} ,$$

23 $$g_{3} \left( {{\varvec{\upsigma}}} \right) = \sigma_{2} - \kappa \,\sigma_{3} ,$$

where *κ* denotes the dilatancy constant:

24 $$\kappa = \frac{1 + \sin \psi }{{1 - \sin \psi }},$$

and $\dot{\lambda }_{\;i}^{vp} \,$ are the viscoplastic multipliers after Perzyna’s theory, which are considered analogously to Zienkiewicz et al. (1975):

25 $$\dot{\lambda }_{i}^{vp} = \frac{{f_{i} \left( {{\varvec{\upsigma}}} \right)}}{\eta }.$$

Using Eq. 25, Eq. 18 can be written as follows (Heeres et al. 2002):

26 $${\mathbf{n}}_{i}^{{\text{T}}} \,{\dot{\mathbf{\sigma }}} - \ddot{\lambda }_{i}^{vp} \,\eta = 0,$$

which evidently reduces to the consistency equation of classic plasticity theory for *η* = 0.

### Stress Integration

The MC-Perzyna model was implemented in Abaqus^®^ (Dassault Systèmes 2018) as a user-defined material subroutine (UMAT) by the first author. At a given iteration *j* + 1, the UMAT: (i) receives as input the stress state ${\tilde{\mathbf{\upsigma}}}_j$ of the previous iteration and the updated strain increment ${\Delta\tilde{{\mathbf\varepsilon}}}_{j+1}$ in the local coordinate system; (ii) transforms them into the principal coordinate system as **σ**_*j*_ and Δ**ε**_*j*+*1*_; (iii) determines the updated stress state **σ**_*j*+*1*_ and assembles the viscoplastic tangent stiffness matrix **D**^*vp*^ = d**σ/**d**ε** in the principal coordinate system, based upon the constitutive equations presented above; and, (iv), returns as output the back-transformed from the principal to the local coordinate system ${\tilde{\mathbf{\upsigma}}}_j$ and $\tilde{\bf D}^{vp}$. The assembly and return of ${\tilde{\mathbf{D}}}^{vp}$ ensures quadratic convergence of the numerical solution algorithm. The algorithmic treatment of the model formulation equations in a discrete form is identical to the one described in Sect. 4.2 of Heeres et al. (2002); the only difference is that the implicit scheme degenerates into an explicit scheme in the present case, due to the planar geometry of the MC yield surface, which enables all quantities to be directly evaluated at the beginning of the iteration.

## List of Symbols

**D**^*el*^: Constant elastic stiffness matrix according to Hooke’s law
**D**^*vp*^: Viscoplastic tangent stiffness matrix in the principal coordinate system
$\tilde{\bf D}^{vp}$: Viscoplastic tangent stiffness matrix in the local coordinate system
*d*_*s*_: Thickness of the shield
*d*_*l*_: Thickness of the lining
*E*: Young’s modulus of the ground
*E*_*c*_: Young’s modulus of the lining
*E*_*s*_: Young’s modulus of the shield
*F*_*r*_: Thrust force required to overcome shield skin friction
*f*_*c*_: Uniaxial compressive strength of the ground
*f*_*i*_: Yield function
*g*_*i*_: Plastic potential function
*H*: Depth of cover
*K*_*s*_: Stiffness of the shield
*K*_*l*_: Stiffness of the lining
*L*: Length of the shield
*L*_*T*_: Length of one lining segment
*m*: Yield surface slope in the principal stress planes
**n**_*i*_: Normal vector to the yield surface *f*_*i*_
*N*: Number of TBM stroke-lining ring erection (“stop-and-go”) cycles
**r**_***i***_: Normal vector to the plastic potential surface *g*_*i*_
*R*: Tunnel radius
*r*: Radial coordinate (distance from the tunnel axis)
*s*: Round length
*t*: Time
*t*_95_: Time to achieve 95% of the time-dependent displacement or lining load increment
*u*: Radial displacement of the ground at the tunnel boundary
v_*N*_: Net advance rate over the TBM strokes
v_*G*_: Gross advance rate over one full excavation cycle
v_*S*_: Smeared advance rate over the “stop-and-go” phase
*x*: Axial coordinate (distance behind the tunnel face)
*x*_*f*_: Distance of the tunnel face from the left model boundary
*γ*_*ij*_: Shear strain component over the plane *ij* of the local coordinate system
Δ**ε**_*j*_: Strain increment in the principal coordinate system at iteration *j*
${\Delta{\tilde{\varepsilon }_j}}$: Strain increment in the local coordinate system at iteration *j*
Δ*T*_1_: Duration of one TBM stroke
Δ*T*_2_: Duration of one lining ring erection
Δ*T*_3_: Duration of TBM standstill following the “stop-and-go” phase
Δ*T*: Duration of one full excavation cycle
Δ*R*: Annular gap (overcut)
**ε**: Strain vector in the principal coordinate system
$\tilde{\varepsilon }$: Strain vector in the local coordinate system
$\dot{\varepsilon }$: Total strain rate vector in the principal coordinate system
$\dot{\varepsilon }^{el}$: Elastic strain rate vector in the principal coordinate system
$\dot{\varepsilon }^{vp}$: Viscoplastic strain rate vector in the principal coordinate system
*ε*_*i*_: Normal strain component along the principal coordinate axis *i*
*ε*_*ii*_: Normal strain component along the local coordinate axis *i*
*ε*^*p*^: Equivalent plastic strain
*η*: Viscosity of the ground
*κ*: Dilatancy constant
$\dot{\lambda }_{i}^{vp}$: Viscoplastic multiplier of plastic potential surface *g*_*i*_
*μ*: Shield skin friction coefficient
*ν*: Poisson’s ratio of the ground
**σ**: Stress vector in the principal coordinate system
$\tilde {\mathbf{\upsigma}}$: Stress vector in the local coordinate system
$\dot{\sigma }$: Stress rate vector in the principal coordinate system
**σ**_*j*_: Stress vector in the principal coordinate system at iteration *j*
$\tilde {\mathbf{\upsigma}_j}$: Stress vector in the local coordinate system at iteration *j*
*σ*_*0*_: in-situ Vertical stress
*σ*_*i*_: Normal stress component along the principal coordinate axis *i*
*σ*_*ii*_: Normal stress component along the local coordinate axis *i*
*σ*_*R*_: Radial ground pressure at the tunnel boundary
$\overline{\sigma }_{R}$: Average radial ground pressure over the shield
*τ*_*ij*_: Shear stress component over the plane *ij* of the local coordinate system
*ϕ*: Angle of internal friction of the ground
*ψ*: Dilatancy angle of the ground
